# A systematic review of the co-occurrence of self-harm and aggression: Is dual harm a unique behavioural construct?

**DOI:** 10.3389/fpsyt.2023.1083271

**Published:** 2023-02-16

**Authors:** Matina Shafti, Peter Taylor, Andrew Forrester, Fritz Handerer, Daniel Pratt

**Affiliations:** ^1^Division of Psychology and Mental Health, School of Health Sciences, Manchester Academic Health Science Centre, The University of Manchester, Manchester, United Kingdom; ^2^Division of Psychological Medicine and Clinical Neurosciences, School of Medicine, Cardiff University, Cardiff, United Kingdom

**Keywords:** dual harm, self-harm, aggression, violence, homicide-suicide, co-occurrence

## Abstract

**Introduction:**

Dual harm is the co-occurrence of self-harm and aggression during an individual’s lifetime. It is unclear whether sufficient evidence exists for dual harm as a unique clinical entity. This systematic review aimed to examine whether there are psychological factors that are uniquely associated with dual harm when compared to those who have engaged in sole harm (self-harm alone, aggression alone) and no harmful behaviours. Our secondary aim was to conduct a critical appraisal of the literature.

**Methods:**

The review searched PsycINFO, PubMed, CINAHL, and EThOS on September 27, 2022, resulting in 31 eligible papers that represented 15,094 individuals. An adapted version of the Agency for Healthcare Research and Quality was used to assess risk of bias and a narrative synthesis was conducted.

**Results:**

The included studies assessed differences in mental health problems, personality, and emotion related factors between the different behavioural groups. We found weak evidence that dual harm is an independent construct with unique psychological characteristics. Rather, our review suggests that dual harm results from the interaction of psychological risk factors that are associated with self-harm and aggression.

**Discussion:**

The critical appraisal identified numerous limitations within the dual harm literature. Clinical implications and recommendations for future research are provided.

**Systematic review registration:**

https://www.crd.york.ac.uk/prospero/display_record.php?RecordID=197323, identifier CRD42020197323.

## 1. Introduction

### 1.1. Background

Self-harm refers to intentional acts of self-injury, irrespective of suicidal or non-suicidal intent (1), whilst aggression is behaviour directed at others with the intention to cause harm (2). When considering their opposing targets, these behaviours appear as two separate constructs. Despite this, research has shown that self-harm and aggression are significantly associated with each other and share risk factors, including early adverse events, problems with emotional functioning and dysfunction of the serotonergic system (3–8). Rather than engage in self-harm *or* aggression, some individuals will engage in both. The co-occurrence of self-harm and aggression during an individual’s lifetime has been referred to as dual harm (9). If dual harm is viewed as a continuum, homicide-suicide may be considered as the most extreme form of this behaviour in regard to the level of harm caused (10), and will therefore be included in our definition of dual harm.

The presence of dual harm has been shown across different ages and populations, including prisoners, psychiatric patients and community samples (8). To our knowledge, the only systematic review that has examined dual harm without solely focussing on homicide-suicide is O’Donnell et al.’s (8) paper. This review found that in the majority of the 23 included studies, the prevalence of aggression in those with a history of self-harm was above 20%. Furthermore, most studies reported a significant positive association between self-harm and aggression (*r* = 0.1–0.6). In 24 studies that had not selected their sample for either harmful behaviour, the prevalence of dual harm was above 15%, with those who had engaged in one of the harmful behaviours being significantly more likely to engage in the other (OR—1.1–38.6, 8). Such findings highlight that not everyone who self-harms is violent (and vice versa), suggesting that those who engage in dual harm represent a distinguishable minority.

Dual harm has especially been reported amongst forensic populations, including prisoners and forensic mental health service users. Studies have reported that up to 56% of these individuals have engaged in both self-harm and aggression (4). As such, dual harm presents a particular concern amongst forensic populations. It is important to extend our understanding of dual harm and the factors associated with this behaviour in order to effectively prevent and reduce this behaviour in prisons and forensic mental health services.

Whilst O’Donnell et al.’s (8) work demonstrates the prevalence of co-occurring self-harm and aggression and their association, it is also important to identify factors that may be linked to dual harm. Hillbrand’s (10) narrative review of 27 papers is the only non-homicide-suicide specific study that aimed to assess factors that could be associated with dual harm. The review highlighted that self-harm and aggression share risk (e.g., sexual abuse) and protective factors, and anger is significantly associated with risk of suicide in violent individuals, thereby implicating such factors in the co-existence of these behaviours (10). Hillbrand’s (10) work highlights that rather than be completely distinct behaviours, self-harm and aggression share various factors that may contribute to their co-occurrence.

Whilst no systematic reviews have directly examined the characteristics associated with less extreme forms of dual harm, there have been a number of reviews that have done so for homicide-suicide. Such reviews have highlighted that compared to suicide alone and/or homicide alone, homicide-suicide perpetrators are more likely to be male, older, and have experienced early adverse events and stressful circumstances prior to the homicide-suicide (11–14). A commonly reported finding is that psychopathology is a risk factor of homicide-suicide, with studies reporting an association between homicide-suicide and mental health service contact and mental health problems, such as depression and personality disorders (11, 13, 14).

The above research highlights that rather than engage in self-harm *or* aggression, some individuals will engage in both. Moreover, these behaviours share risk factors and are significantly associated with each other. In light of such findings, rather than exclusively distinguish between self-harm and aggression, it is important to consider why individuals may engage in dual harm and the factors that may underlie this behaviour. Despite this, given the long-standing distinction made between self-harm and aggression within research and practice, our understanding of dual harm is limited. At the government level, aggression is primarily managed within the criminal justice system, whilst self-harm is typically managed within the mental health system (9). Moreover, there are currently no established clinical guidelines for how to manage dual harm within forensic (e.g., prisons, forensic psychiatric services) and clinical (e.g., psychiatric hospitals) settings (15). This may be concerning given reports that prisoners who have engaged in dual harm are significantly more likely to be in disciplinary programmes and spend longer in prison compared to those who have engaged in aggression alone (9). Such evidence suggests that our current approach towards dual harm is insufficient. In order to effectively prevent and manage co-occurring self-harm and aggression, it is imperative to develop our understanding of the mechanisms that may contribute to this behaviour.

### 1.2. Theories of dual harm

The literature around homicide-suicide and less extreme forms of dual harm has largely been separate, with theories of dual harm primarily emerging from the former. Homicide-suicide has largely been accounted for within existing theories of harmful behaviours, including psychodynamic (16), attribution (17), strain (18), and social integration theories (19, 20) [see Liem (11) for a full discussion]. These theories tend to explain homicide-suicide within a primarily suicide or aggression driven framework, reflecting the debate as to whether homicide-suicide is primarily motivated by homicidal or suicidal intent (21). For example, Durkheim (19) adopted a sociological framework to account for the relationship between self-harm and aggression using social integration theory. Social integration theory suggests that homicide and suicide are linked and driven by similar social mechanisms, including social organisation and integration within various communities in society. The rate of suicide within a society increases when an individual’s relationship with their society is weak. Homicide-suicide is perceived as an extreme form of suicidal behaviour that occurs as a result of extensive social disintegration. Support for the social integration theory is provided by evidence that social isolation increases the likelihood of homicide-suicide (20, 22). However, research has shown that those who engage in suicide alone are significantly more likely to be socially isolated than those who have engaged in homicide-suicide, suggesting that social disintegration may not be the primary driving force of homicide-suicide (23).

Rather than be considered within a primarily self-harm or aggression framework, other theories have suggested that these behaviours co-occur due to a shared underlying aetiology. For example, within a psychoanalysis framework, self-harm has been viewed as violence turned inwards, in which a shared aggressive drive underlies both of these behaviours (24, 25). Similarly, the stream analogy of homicide-suicide suggests that homicide and suicide emerge from a single stream of violence. Here, social and cultural forces of direction influence whether an individual attributes the blame of their frustration towards themselves (internal attribution) or others (outwards attribution). While external attributions increase the risk of homicide, internal attributions increase the risk of suicide (24). External attributions may be driven by perceived discriminatory deprivation and social subordination, in which an individual or group has an inferior position within the social hierarchy and blames their problems on such injustice. Internal attributions can include factors such as economic development, in which the individual may feel more in control of their outcomes and thus blame themselves for their problems (26). It is suggested that when both an inward and outward attribution for frustration exists, this increases the risk of homicide-suicide (21, 27, 28).

In keeping with the suggestion that self-harm and aggression are driven by shared mechanisms, Plutchik and Van Praag’s (29) two-stage model of countervailing forces posits that an underlying aggressive impulse leads to both self-harm and aggression. In the first stage of the model, triggers (e.g., threat, loss of control) lead to an aggressive impulse that is then amplified or weakened depending on the presence or absence of certain factors. The interaction of such factors determines the likelihood of harmful behaviours. In the second stage, countervailing factors influence the object of the behaviour—self vs. the other. These factors are based on Plutchik and Van Praag’s (29) research where it was found that specific variables, such as depression, hopelessness, and psychiatric symptoms, increase an individual’s risk of directing their aggressive impulse towards themselves. On the other hand, factors such as impulsivity, recent life stresses and psychopathy were shown to increase the likelihood of the impulse being directed towards others (10). Hillbrand (10) suggests that in the context of Plutchik and Van Praag’s (29) model, the presence of both sets of factors would increase an individual’s risk of dual harm. As well as having a common underlying aggressive drive, co-occurring self-harm and aggression has been suggested to be driven by other shared factors, such as impulsivity, lack of behavioural control and emotional dysregulation (8, 26–28).

To the best of our knowledge Shafti et al.’s cognitive-emotional model (15, 30) provides the only comprehensive framework that accounts for how various factors may interact to lead to dual harm and the function of this behaviour. Self-harm and aggression are suggested to not only share a causal pathway, but also serve the same purpose in those who dual harm. In the distal stage, biological and environmental factors combine to develop a personality style that makes an individual vulnerable to harmful behaviours. Subsequently, in the proximal stage, this personality style predisposes the individual to emotional and interpersonal problems that increase their likelihood of engaging in both self-harm and aggression as a way to regulate their negative emotions. On the other hand, dual harm may also serve an interpersonal purpose, such as establishing autonomy. It is the social context and situation that an individual is in, combined with their expectancies, that interact to influence the specific function and behaviour that the individual chooses to engage in at a specific point of time (15).

#### 1.2.1. Dual harm—A unique behavioural construct?

There is growing evidence that compared to those who have engaged in self-harm alone or aggression alone (i.e., sole harm), individuals with a history of dual harm show more frequent, severe (e.g., overdose, hanging) and wider range of harmful and antisocial behaviours (31–35). For example, despite representing a minority within the prison population, prisoners who have engaged in dual harm have been found to be responsible for twice as many incidents of misconducts compared to those who have engaged in sole harm (33). Moreover, compared to sole harm behaviours, there is evidence that dual harm is significantly more likely to be associated with various adverse social, environmental and psychological factors, including childhood polyvictimisation, substance use, childhood abuse, low self-control, difficulties with self-regulation, and psychopathy (35–38). The above research highlights that compared to those who sole harm, individuals that engage in dual harm show a greater level of risk across a range of factors, thereby representing a riskier group.

**FIGURE 1:**
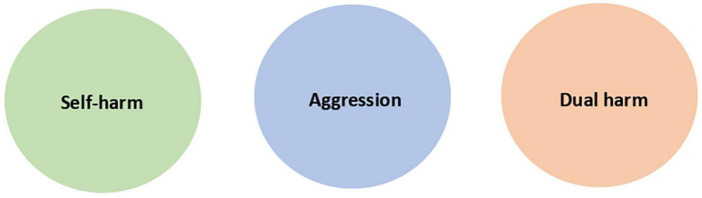
Dual harm as a unique behavioural construct.

In light of such findings, it has been suggested that dual harm is a unique phenomenon that cannot be “reduced to a sum of its components” (9, 11, 12, 21, p. 1,182). In that, rather than be an overlap between self-harm and aggression, dual harm is as an independent behavioural construct with characteristics that make it unique from sole harm behaviours (Figure 1). If this is the case, it would be important to develop tailored interventions that target the distinct aspects of dual harm behaviour.

**FIGURE 2:**
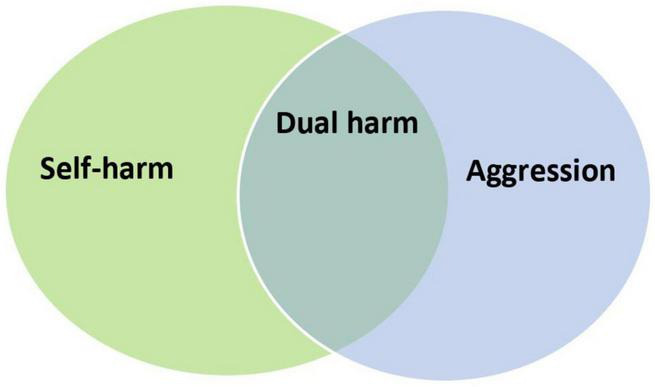
Dual harm as an overlap between self-harm and aggression.

However, at this stage, it is unclear whether it is meaningful to approach dual harm as a unique behavioural construct with distinct characteristics. Although there is evidence that those who engage in dual harm are significantly more likely to present with various factors compared to individuals who sole harm, this does not necessarily mean that these factors are *unique* to dual harm. Conversely, it may be that these factors are separately linked to self-harm and aggression, and it is their interaction and multiplicative effect that lead to dual harm and the riskier profile shown by these individuals. In line with this, Boxer (4) suggested that co-occurring self-harm and aggression results from *a* “*high loading*” of risk across various personal and situational factors related to harmful behaviours. Accordingly, rather than be a unique behavioural construct, dual harm may develop as a result of the overlap between self-harm and aggression and their risk factors (Figure 2). In this case, it would be crucial for researchers and clinicians to adopt an integrated approach that considers the factors associated with self-harm and aggression together in the context of dual harm.

### 1.3. This systematic review

Despite the historic separation between self-harm and aggression, previous research has demonstrated the importance of considering these behaviours together in the context of dual harm. However, we still have limited understanding of dual harm and how to approach this behaviour within both research and clinical practice. It is unclear whether dual harm should be considered as a unique behavioural construct with distinct characteristics when compared to sole harm behaviours. Identifying how dual harm is most meaningfully understood is imperative in the effective management and intervention of this behaviour within forensic and clinical settings.

Therefore, we aimed to conduct the first systematic review that addresses the following question: *compared to those who have engaged in self-harm alone, aggression alone, and no harmful behaviours, are there psychological factors that are uniquely associated with those who have engaged in dual harm?* We focus on the role of psychological factors as these may be more modifiable through intervention than social, environmental and biological factors, and thus allow us to provide greater practical implications. Evidence that specific psychological factors are associated with dual harm when compared to *all* other behavioural groups (i.e., self-harm alone, aggression alone, no harmful behaviours) would support the notion that dual harm is a unique clinical construct with distinct characteristics. The secondary aim of this review was to evaluate the methodological strengths and weaknesses of the included literature to inform future studies of dual harm. Our work builds on previous empirical studies and reviews in order to extend our understanding of dual harm and the characteristics of this behaviour.

## 2. Methods

This systematic review was carried out in line with PRISMA 2020 guidelines using the PRISMA 2020 checklist (Supplementary Appendix A; 39). As is best practice (40), the protocol for this review was pre-registered and is available on PROSPERO (title: A systematic review of the co-occurrence of self-harm and violence: Is dual harm a unique behavioural construct? [CRD42020197323]): https://www.crd.york.ac.uk/prospero/display_record.php?RecordID=197323.

The first version of the protocol for this systematic review was amended. We first intended to assess sociodemographic, psychological, and environmental characteristics that may be uniquely associated with dual harm. However, after our scoping review, we discovered that examining all of these factors would over-extend the scope of this review. We chose to focus on psychological factors as these are more modifiable with intervention. Furthermore, we made an amendment to explicitly state the exclusion of individuals with developmental conditions as their harmful behaviours may be a direct consequence of such conditions and be associated with distinct factors.

### 2.1. Search strategy

On September 29, 2022, we searched the literature within the PsycINFO, PubMed, CINAHL databases, as well as EThOS for theses. These databases were chosen based on the topic area of psychiatry and psychology and previous systematic reviews of dual harm (8, 13). The search was not restricted to time of publication, however, it was limited to papers written in English and human studies.

The following search terms and Boolean operators were used:

(“self-harm*” OR “self-injur*” OR “suicid*” OR “DSH” OR “NSSI”) AND (“violen*” OR “aggressi*”). DSH refers to deliberate self-harm, while NSSI refers to non-suicidal self-injury.

We also searched reference lists of eligible articles and key reviews of dual harm, carried out forward citation searching and contacted authors of eligible papers to inquire about other relevant work. The search further included grey literature by looking for dissertations and theses, and contacting authors of any identified conference abstracts about related papers.

### 2.2. Inclusion and exclusion criteria

We used the Population, Intervention, Comparison, Outcome, Study (PICOS; 41) design framework in Table 1 to inform our inclusion and exclusion criteria for all peer-reviewed papers and theses. Papers and theses that only presented descriptive findings (i.e., summarising characteristics of the outcome) were excluded to allow more meaningful data interpretations. We focused the review on the adult population as studies have reported age to be linked to a distinct pattern of harmful behaviours and risk factors, suggesting developmental differences in the factors associated with dual harm (42–44). Moreover, given that dual harm has been shown to be a particular concern amongst the adult forensic population, this review aimed to highlight psychological factors that may be amenable to treatment within this group. The practical implications for supporting younger populations who dual harm are distinct (e.g., interventions in school, family environment), and therefore, warrants a separate review. Original quantitative research articles published in peer-reviewed journals and dissertations were eligible. Further exclusion criteria included articles in which the analysis did not provide new findings relevant to our review question, letters, newsletters, and book chapters.

**TABLE 1:** PICOS framework.

Search domains	Inclusion criteria	Exclusion criteria
Population	● Individuals with a history of both self-harm and aggressive behaviour ● Mean sample age 18 years old or over, or minimum age 18 if mean not reported ● If longitudinal study, data was collected from participants when they were 18 years old or over	● Sample has developmental condition
Intervention (exposure)	● Psychological factors in relation to dual harm status	
Comparator	● Comparator group included those with a history of self-harm alone, aggression alone, or no harmful behaviours ● Differences in psychological factors reported between participants in the dual harm group and comparator groups	
Outcome	● Harmful behaviour status, e.g., dual harm, self-harm alone, aggression alone, neither harmful behaviour ● All definitions of self-harm and aggressive behaviours	● Ideation of harmful behaviours ● Does not report dual harm as an outcome
Study design	● Quantitative designs ● Mixed methods where the quantitative data is relevant to the review	● Qualitative designs ● Case studies ● Only descriptive findings reported

Psychological factors were defined as variables relating to affective, psychological and cognitive functioning that may be modifiable with psychological intervention. In regard to dual harm, it has not been established which specific forms of self-harm and aggression encompass this behaviour, or how close in time these harmful behaviours should occur in relation to each other. In line with current definitions, participants in the dual harm group were individuals who had engaged at any point during their lifetime in both self-harm and aggressive behaviour, regardless of intent (e.g., suicidal, homicidal) and outcome (e.g., injury or death). No restrictions were placed on the timeframe in which these behaviours must have co-occurred. Ideation of harmful behaviours was not included as research has found differences in factors associated with harmful ideations and behaviours (44). As mentioned, this review includes homicide-suicide in its definition of dual harm. An act tends to be considered as homicide-suicide if the homicidal and suicidal behaviour occur within 24 h. However, some studies extend this time-period, whilst others do not specify one at all (14). Given such inconsistencies in the literature, this review considered an act as homicide-suicide if the researchers defined it as so.

There is variability as to how self-harm and aggression are defined within the literature. Some researchers distinguish between suicidal and non-suicidal intent, whilst others do not assess intent at all (45). Furthermore, outcomes of self-harm differ in lethality, ranging from minor harm, severe harm and in extreme cases, death (i.e., suicide). Likewise, aggression can range from minor acts (e.g., verbal aggression, property damage) to more severe acts (e.g., physical fighting), and in extreme cases, homicide. Given that the literature has not restricted dual harm to intent or severity, this work will not limit the definition of self-harm and aggression to such criteria. Therefore, aggression is defined as any type of aggressive behaviour towards any target (e.g., property, person, verbal, physical), whilst self-harm refers to “intentional acts of self-poisoning or self-injury irrespective of motivation” (46, p. 255). All aggression and self-harm measures were eligible if they recorded behaviours that met the above descriptions.

### 2.3. Screening

The search results were exported and stored onto a reference management software—EndNote version X9 (Thomson Reuters, New York, NY, USA), which was used to remove duplicate references. The lead reviewer (MS) screened all titles and abstracts, then screened the full text of remaining articles. Articles that were not eligible were excluded. To ensure inter-rater reliability at both the title/abstract and full-text levels, a second independent reviewer screened a random sample of 10% of papers. Any disagreements between the reviewers were resolved by consultation with the research team. At the title/abstract and full text levels, inter-rater reliability was 98.5 and 95.2%, respectively, showing almost perfect agreement (Cohen’s *k* = 0.94, *p* < 0.001, Cohen’s *k* = 0.90, *p* < 0.001, respectively).

### 2.4. Data extraction

A data extraction form was developed to identify and extract data across the studies using a standardised method. A second reviewer independently checked the extracted data for errors. All inferential results regarding differences in psychological factors between those who had engaged in dual harm and sole harm behaviours or no harmful behaviours were extracted. Furthermore, we extracted data about the study location, design, relevant groups of study, number of participants, age and sex of participants, and the harmful behaviours and psychological factors examined. The relevant extracted data were entered into a table (Supplementary Appendix B).

### 2.5. Risk of bias (quality) assessment

An adapted version of the Agency for Healthcare Research and Quality (47; Supplementary Appendix C) was used to examine the risk of bias in the included articles. This tool has been used in previous systematic reviews of harmful behaviours (48, 49) and has been designed to be adapted to the review being carried out, as has been done in previous research (47). The Agency for Healthcare Research and Quality identifies the risk of bias in each study by examining the extent to which they meet key methodological criteria. In line with previous research, a summary rating was provided to demonstrate the total risk of bias present in each paper (50). A study was rated as having a high risk of bias if it fully met 0–2 criteria, moderate risk if it fully met 3–5 criteria and low risk if it fully met 6+ criteria (indicated by the number of “yes” ratings; 50). A second reviewer independently conducted the risk of bias assessment for all included papers and disagreements were resolved by discussion with the research team.

### 2.6. Reporting

In line with previous reviews of dual harm (8, 10), given that definitions and measurements of harmful behaviours vary considerably in the literature, it was decided that a meta-analysis would not be appropriate. Therefore, a narrative synthesis of the included papers was conducted according to principles from the Economic and Social Research Council’s guidance for narrative reviews (51). This included developing a preliminary synthesis of findings, exploring relationships in the data and assessing the robustness of the synthesis. Following such principles provided a systematic and transparent synthesis of the included literature. In this synthesis, relevant statistics provided by each study (i.e., effect size, prevalence rates) are reported. In cases where relevant data was missing (e.g., summary statistics), authors were contacted to ask about such data.

The literature of homicide-suicide and less extreme forms of dual harm has largely been separate and at the current stage, it is unclear whether it is meaningful to divert from this separation and examine these behaviours together as one construct. Given the conceptual, theoretical and methodological differences in how these behaviours have been approached, the authors decided to categorise the current synthesis into homicide-suicide and non-homicide-suicide research. For example, in order to be considered as homicide-suicide, the self-harm and aggressive acts must co-occur within a short time-period and by definition, must constitute the most lethal forms of these behaviours (i.e., homicide and suicide). However, these restrictions have not been placed in conceptualisations of non-homicide-suicide dual harm. Furthermore, given that homicide-suicide is a rare event, studies generally tend to use large national databases to identify these cases. This is distinct from less extreme forms of dual harm in which a range of measures have been used to assess this behaviour, including questionnaires, interviews and official records.

## 3. Results

**FIGURE 3:**
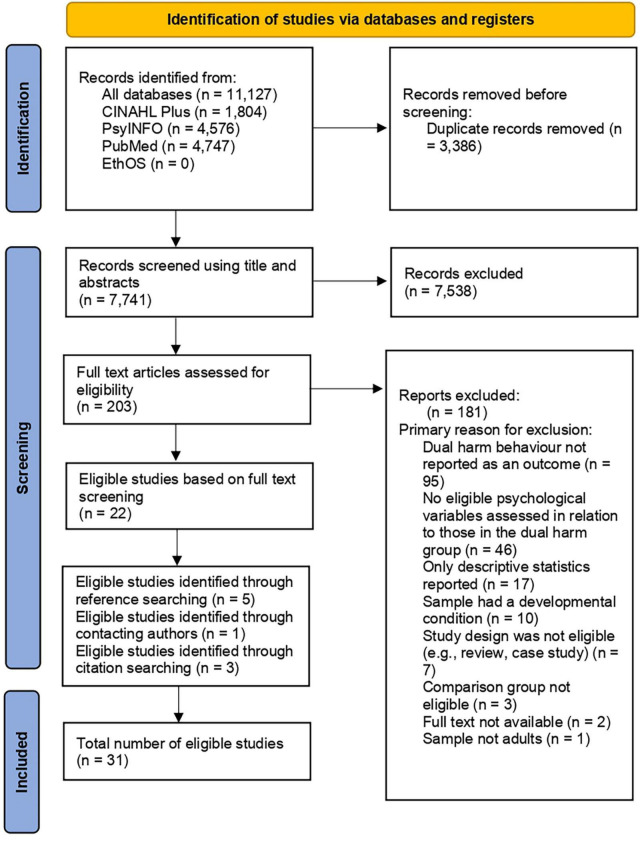
PRISMA flowchart.

As recommended by PRISMA guidelines (39), the search process for this systematic review is demonstrated using the PRISMA flowchart (Figure 3). Harford et al.’s (52) paper appeared to meet the inclusion criteria. However, upon reading the full text, we found that ideation was included in their self-harm measure. Since it was not clear whether the dual harm and self-harm alone group included those who had indicated to have engaged in self-harm ideation but not behaviour, we excluded the above paper. The excluded articles and the primary reason for their exclusion at the full text screening level is outlined in Supplementary Appendix D.

### 3.1. Study characteristics

Tables 2, 3 show the summary characteristics of the included studies. Only information relevant to this review are reported. Fifteen studies focussed on homicide-suicide, whilst sixteen examined less extreme forms of dual harm. In total, there was 15,094 participants in the included studies, 9,875 of which were from homicide-suicide studies and 5,219 from the non-homicide-suicide literature. The sample size of the dual harm groups ranged from 22 to 2,535 in homicide-suicide studies (23), and 11 to 1,060 in non-homicide-suicide research (38, 53). The included literature was conducted in nine reported locations, mostly North America and Europe. Hillbrand’s (31) study did not report a location, but it was inferred that the research was conducted in the USA as the author was based there during the time of the study.

**TABLE 2:** Summary characteristics of homicide-suicide studies.

References	Country	Relevant groups of study–N of participants	Harmful behaviours examined: measure(s)	Psychological factor(s) examined: measure(s)	Reported findings
Benítez-Borrego et al. (64)	Chile	1: Filicide-suicide perpetrators–33 2: Filicide alone perpetrators–35	Filicide-suicide and filicide alone: cases reported in the Legal Medical Service	Diagnosed psychiatric symptoms: forensic reports from forensic psychiatric and psychological evaluations in Legal Medical Service	No significant differences in diagnosed psychiatric symptoms
Flynn et al. (76)	England and Wales	1: Homicide-suicide perpetrators–203 2: Those who had engaged in suicide alone–46,358 3: Homicide alone perpetrators–5,096	Homicide alone, completed suicide, and homicide-suicide: case records from Home Office and police	Mental health problems: questionnaire completed by individual’s mental health team	Compared to homicide-suicide perpetrators, those who engaged in suicide alone were significantly more likely to have a severe mental illness, while homicide-suicide perpetrators were significantly more likely to have a personality disorder No significant differences between homicide alone and homicide-suicide groups in schizophrenia, affective disorder, and personality disorder
Fridel and Zimmerman (65)	USA	1: Homicide-suicide perpetrators–2,048 2: Those who had engaged in suicide alone–103,195	Suicide alone and homicide-suicide: cases of deaths reported by the National Violent Death Reporting System (NVDRS) in 2003–2013	Mental health stressors: coroner/medical examiner reports and law enforcement reports in the NVDRS	All mental health stressors, including depressed mood, were significantly more prevalent amongst those who had engaged in suicide alone than homicide-suicide perpetrators
Fridel and Zimmerman (65)	USA	1: Homicide-suicide perpetrators–1,413 2: Those who had engaged in suicide alone–81,179 3: Homicide alone perpetrators–22,960	Homicide-suicide, homicide alone, suicide alone: cases of deaths reported by NVDRS in 2003–2013	Mental health: coroner/medical examiner records and law enforcement reports in the NVDRS	Homicide-suicide perpetrators were significantly more likely to have mental health problems than homicide alone perpetrators, with the risk of a suicide after homicide increasing for those with mental health problems Those who engaged in suicide alone were significantly more likely to have mental health problems than homicide-suicide perpetrators
Friedman et al. (65)	USA	1: Mothers who had perpetrated filicide followed by non-fatal and fatal suicide–29 2: Mothers who had perpetrated filicide alone–20	Filicide-suicide and filicide alone: records from coroner’s office	Psychotic symptoms and depression: not specified	Compared to filicide alone and filicide-attempted suicide, mothers who engaged in filicide-completed suicide were significantly less likely to have been noted to be delusional. There were no significant differences in depression, auditory hallucinations, or command auditory hallucinations
Haines et al. (23)	Tasmania	1: Homicide-suicide perpetrators–22 2: Those who had engaged in suicide alone–22	Suicide and homicide-suicide: files from coroners at the Tasmanian Archives Office and Department of Justice	Psychological symptoms: files from coroners	There was a trend for fewer of the homicide-suicide group to have experienced anxiety in the time leading up to their death
Heron (70)	Canada	1: Intimate homicide-suicide perpetrators–64 2: Intimate homicide alone perpetrators–158	Intimate homicide-suicide and intimate homicide alone: case records from the Ontario Domestic Violence Death Review Committee (DVDRC)	Depression and other mental health problems: records from Ontario DVDRC. Diagnosis of depression based on the opinion of professionals and non-professionals	There was a significantly larger amount of homicide-suicide perpetrators who had been professionally and unprofessionally diagnosed with depression than homicide alone perpetrators Homicide-suicide and homicide alone perpetrators did not significantly differ based on other psychiatric diagnoses
Kalesan et al. (66)	USA	1: Homicide-suicide perpetrators–1,422 2: Those who had engaged in suicide alone–41,244	Homicide-suicide and suicide alone: cases of deaths reported by the NVDRS in 2003–2011	Depression and mental health issues: NVDRS records	Across all ages, depression decreased risk of homicide-suicide with a firearm compared to suicide alone Depression and mental health issues decreased risk of homicide-suicide compared to suicide alone in those under 30 years old and over 30 years old
Leveillee et al. (62)	Canada	1: Filicide-suicide perpetrators–38 2: Filicide alone perpetrators–37	Filicide-suicide and filicide alone: files of cases compiled by the Bureu du Coroner en Chef du Quebec	Depressive and psychotic disorders: coroner’s reports, psychiatric and medical records, summaries of investigations by the Youth Protection Commission and the Youth Protection Directorate	Males who engaged in filicide-suicide were significantly more likely than those who engaged in filicide alone to have depressive disorders
Liem et al. (67)	Netherlands	1: Homicide-parasuicide perpetrators–77 2: Homicide alone perpetrators–430 3: Those who had engaged in parasuicide alone–160	Homicide-parasuicide and homicide alone: cases reported in a forensic psychiatric hospital Parasuicide alone—cases reported in a psychiatric hospital. Classified as a parasuicide using the Pierce Suicide Intent Scale	Psychopathological characteristic based on DSM-IV: case files in psychiatric hospital. If diagnosis not in files, a retrospective diagnosis was made according to file information	Homicide–parasuicide perpetrators were significantly more likely than homicide alone perpetrators to have a mood disorder, most notably depression. Depression raised the odds of a parasuicide following a homicide more than 15 times. However, there were no differences in psychotic disorder or personality disorder. Homicide-parasuicide perpetrators were significantly more likely to be diagnosed with a psychotic disorder and personality disorder compared to the parasuicide alone group. However, there were no significant differences in mood disorders
Liem and Roberts (25)	Netherlands	1: Intimate homicide-suicide perpetrators–44 2: Intimate homicide alone perpetrators–297	Intimate homicide-suicide and intimate homicide alone: archive of clinical records in a forensic psychiatric hospital	Psychopathology: case records from forensic psychiatric hospital	The homicide-suicide and homicide alone groups did not significantly differ in psychotic disorders Homicide-suicide perpetrators were significantly more likely to be diagnosed with a depressive disorder
Logan et al. (73)	USA	1: Homicide-suicide perpetrators–408 2: Those who had engaged in suicide alone–20,183	Homicide-suicide and suicide alone: cases of deaths reported by the NVDRS in 2003–2005	Mental health problems, depressed mood: NVDRS records	Compared with males who engaged in suicide alone, male homicide-suicide perpetrators were significantly less likely to have reports of depressed mood and mental health problems
Logan et al. (72)	USA	1: Intimate homicide-suicide perpetrators–1,504 2: Those who had engaged in suicide alone–28,755	Homicide-suicide and completed suicide alone: cases of deaths reported by the NVDRS in 2003–2015	Current depressed mood and mental health condition: NVDRS records	Intimate homicide perpetration was less prevalent among suicide decedents who had a known current depressed mood and mental health conditions
Vatnar et al. (63)	Norway	1: Intimate homicide-suicide perpetrators–44 2: Intimate homicide alone perpetrators–133	Homicide alone and homicide-suicide: cases identified from the Norway Criminal Investigation Service (NCIS)	Professionally diagnosed mental health diagnosis: reports from the NCIS statistics	No significant differences in mental health diagnosis
Zimmerman and Fridel (71)	USA	1: Homicide-suicide perpetrators–2,535 2: Homicide alone perpetrators–28,027 3: Those who had engaged in suicide alone–138,948	Homicide-suicide, homicide alone, and completed suicide: cases of deaths reported by the NVDRS in 2003–2015	Mental health problems: NVDRS records	Odds of suicide following homicide were significantly elevated for perpetrators with mental health problems

**TABLE 3:** Summary characteristics of non-homicide-suicide studies.

References	Country	Relevant groups of study–N of participants	Harmful behaviours examined: measure(s)	Psychological factor(s) examined: measure(s)	Reported findings
Ghossoub et al. (55)	USA	Nationally representative sample of non-institutionalised, household-based civilian population from the National Survey on Drug Use and Health (NSDUH): 1: Those who had engaged in dual harm–410 2: Those who had engaged in no harmful behaviours–259,914	Past year suicidal behaviour and physical attacks towards others: based on self-reported answers to two questions Dual harm: cross-tabulation of responses to the above measures	Past year substance use disorder and psychiatric disorder: self-reported survey based on DSM-IV criteria	Compared to those who had no history of harmful behaviours, substance use disorders and psychiatric disorders were significantly more prevalent in the dual harm group Alcohol use disorders, drug use disorders, and alcohol and drug use disorders significantly increased the odds of perpetrating dual harm compared to having no history of harmful behaviours, even after adjusting for sociodemographic characteristics
Harford et al. (75)	USA	Civilian non-institutionalised population from the National Epidemiological Survey on Alcohol and Related Conditions (NESARC): 1: Those who had engaged in dual harm–688 2: Those who had engaged in aggression alone–4,689 3: Those who had engaged in self-harm alone–996	Lifetime physical aggression towards others: bespoke self-report questionnaire of five items Suicidal behaviour: based on one question asking about lifetime suicidal attempt and one question asking about suicidal behaviour in those who screened positive for a DSM-IV major depressive episode Dual harm: cross-tabulation of responses to the above measures	Lifetime DSM-IV diagnosis of psychiatric disorders: Alcohol Use Disorder and Associated Disabilities Interview Schedule	Odds of substance use disorder was significantly higher for dual harm group compared to self-harm alone group Odds of personality disorder was significantly higher for dual harm group compared to self-harm alone and aggression alone group Odds of mood disorders was significantly higher for dual harm group compared to self-harm alone and aggression alone group Odds of anxiety disorders was significantly higher for dual harm group compared to aggression alone group
Harford et al. (53)	USA	Civilian non-institutionalised population from NESARC-III: 1: Those who had engaged in dual harm–1,060 2: Those who had engaged in aggression alone–4,038 3: Those who had engaged in self-harm alone–1,730 4: Those who had engaged in no harmful behaviours–29,481	Suicidal behaviour: one question asking about lifetime suicidal attempt and one question asking about suicidal behaviour in preceding 2 weeks during the time they experienced depression or mania Aggression: had engaged in at least one of seven aggressive behaviours since age of 15. Not specified whether these questions were self-reported Dual harm: cross-tabulation of responses to the above measures	Lifetime DSM-IV diagnosis of psychiatric disorders: Alcohol Use Disorder and Associated Disabilities Interview Schedule	Substance use disorder, bipolar 1 disorder, panic disorder, generalised anxiety disorder, post-traumatic stress disorder, schizotypal personality disorder, antisocial personality disorder, and borderline personality disorder were significantly associated with higher odds for dual harm relative to aggression alone and self-harm alone When adjusting for sociodemographic characteristics and lifetime DSM-5 disorders–all substance use disorders showed significantly higher odds for dual harm relative to no history of harmful behaviours. Alcohol, tobacco, and other drug use disorders showed significantly higher odds for dual harm relative to self-harm alone. Mood disorders showed significantly higher odds for dual harm relative to history of no harmful behaviours and aggression alone. Post-traumatic stress disorder, schizotypal personality disorder, antisocial personality disorder, and borderline personality disorder showed significantly higher odds for dual harm relative to no history of harmful behaviours. Antisocial personality disorder and borderline personality disorder also had significantly higher odds for dual harm relative to self-harm alone, as did borderline personality disorder for dual harm relative to aggression alone
Harford (58)	USA	Nationally representative sample of non-institutionalised, household-based civilian population from NSDUH: 1: Those who had engaged in dual harm–464 2: Those who had engaged in self-harm alone–2,289 3: Those who had engaged in aggression alone–7,286 4: Those who had engaged in no harmful behaviours–304,842	Past year suicidal behaviour and physical attacks towards others: based on self-reported answers to two questions Dual harm: cross-tabulation of responses to the above measures	Substance use disorders: based on DSM-IV diagnoses, but not reported how this was assessed Nicotine dependence: Nicotine Dependence Syndrome Scale and the Fagerstrom Test of Nicotine Dependence Serious psychological distress: Kessler-6	Compared with the self-harm alone, aggression alone, and no harmful behaviours groups, the dual harm group were significantly more likely to have serious psychological distress, nicotine dependence and four or more DSM-IV SUD criteria for alcohol, cocaine, pain reliever, and stimulant use disorders Compared to self-harm alone and no harmful behaviours groups, the dual harm group was significantly more likely to have four or more DSM-IV marijuana use disorder criteria
Hemming et al. (54)	UK	1: Prisoners who had engaged in dual harm–12 2: Prisoners who had engaged in self-harm alone–4 3: Prisoners who had engaged in aggression alone–25	Aggression assessed over past 2 weeks: bespoke five item questionnaire Suicide alone assessed over past 2 weeks: bespoke six item questionnaire Dual harm: cross-tabulation of responses to the above measures	Alexithymia: Toronto Alexithymia Scale Anger: The Novaco Anger Scale Impulsivity: The Dickman Impulsivity Inventory	No significant differences in alexithymia, anger, or impulsivity
Hillbrand (31)	Not reported (assumed USA)	Forensic psychiatric patients with a history of severe violence: 1: Those who had engaged in aggression alone–35 2: Those who had engaged in dual harm–15	Self-harm irrespective of suicidal intent and aggression during a 6 months period: Overt aggression scale Dual harm: cross-tabulation of responses to the above measures	Psychiatric diagnosis: medical records	No significant differences in personality disorders, alcohol/substance abuse or psychotic disorders
Huang et al. (37)	China	Individuals with serious aggressive behaviours and suspected mental disorder in seven forensic institutes in different provinces 1. Those who had engaged in dual harm–74 2. Those who had engaged in aggression alone–349	Lifetime self-harm (unclear if non-suicidal self-harm assessed): self-report questionnaire Serious aggressive behaviours: participants’ forensic archives Dual harm: cross-tabulation of responses to the above measures	History of substance abuse and mental disorders: standardised data collection form and forensic archives Current mental disorder: evaluated by two psychiatrists using ICD-10 Psychopathy: Chinese version of Psychopathy Checklist-Revised (PCL-R) Psychiatric symptoms: Chinese version of Brief Psychiatric Rating Scale (BPRS)	Compared to the aggression alone group, the dual harm group were significantly more likely to have a history of mental disorder, current mental disorder, score higher on the anti-social scale of the PCL-R, and score higher on the anxiety-depression scale of the BPRD. There were no significant differences in substance use.
Laporte et al. (56)	Sweden	Young adult violent offenders: 1: Those who had engaged in dual harm–62 2: Those who had engaged in aggression alone–208	Lifetime suicidal and non-suicidal self-harm: files and interviews Dual harm: based on responses to above measure	Mental disorder: Structured Clinical Interview guides for Axis I and II disorders and file information Symptoms of autism spectrum disorders and other neurodevelopmental disorders: Asperger syndrome/high functioning autism diagnostic interview and structured DSM-IV interview protocol	The dual harm group had significantly more childhood attention deficit symptoms, adult attention deficit symptoms and adult hyperactivity disorder symptoms than the aggression alone group. There was no significant difference in childhood hyperactivity disorder symptoms
Lidberg et al. (61)	Sweden	Male homicide offenders: 1: Those who had engaged in dual harm–12 2: Those who had perpetrated homicide alone–23	Suicide attempts: forensic psychiatric reports Dual harm: cross-tabulation of responses to the above measure	Personality: The Eysenck Personality Inventory, Eysenck Personality Questionnaire, Marke-Nyman Temperament Scale, Gough Delinquency Scale	There were no significant differences in personality
Richmond-Rakerd et al. (35)	UK	Twins of the E-Risk Longitudinal Twin Study: 1: Those who had engaged in dual harm–97 2: Those who had engaged in self-harm alone–177 3: Those who had engaged in aggression alone–not reported 4: Those who had engaged in no harmful behaviours–1,475	Self-harm, irrespective of suicidal intent: life history calendar used to aid recall of self-reported self-harm behaviour since age 12 Aggression: official police records and self-report questionnaire assessing past-year violent offending behaviour Dual harm: cross-tabulation of responses to the above measures	Mental health difficulties: DSM-IV based symptoms/diagnosis of post-traumatic stress disorder, depression, psychosis, and substance dependence. No information on how this data was collected. Personality: Big Five Inventory Self-regulation: Shedler–Westen Assessment Procedure 200-item Q-Sort for Adolescents and an unvalidated questionnaire Self-control: Based on 9 measures, including observational ratings, parent and teacher reports, self-reports, and interview judgements	The dual harm group did not significantly differ from the self-harm alone group in childhood depression, childhood anxiety, or risk of developing post-traumatic stress disorder or depression. However, they were distinguished by a significantly higher prevalence of psychotic symptoms and more likely to meet criteria for alcohol and cannabis dependence Compared to the aggression alone group, the dual harm group exhibited significantly higher rates of childhood depression and all adolescent mental health difficulties Low childhood self-control significantly increased odds of engaging in dual harm compared to those who engaged in self-harm alone. Children rated as having more self-regulation difficulties were significantly more likely to be in the dual harm group than the self-harm alone group Compared to the self-harm alone group, the dual harm had significantly lower openness, conscientiousness, and agreeableness. They were also significantly higher on extraversion Compared to the no harmful behaviours group, the dual harm group were significantly higher on neuroticism and lower in conscientiousness and agreeableness Compared to the aggression alone group, the dual harm group were significantly lower in conscientiousness and higher in neuroticism
Stålenheim (38)	Sweden	Forensic psychiatric male patients: 1: Those who had engaged in dual harm–11 2. Those who had engaged in self-harm alone–12 3: Those who had engaged in aggression alone–15 4: Those who had engaged in no harmful behaviours–20	Suicidal behaviour: based on SCID interviews and filed information from the forensic psychiatric assessments. Repeated violent criminality: identified from participants’ registered violent criminality. Dual harm: cross-tabulation of responses to the above measures	Personality: Karolinska Scales of Personality	No significant differences in psychopathy and aggression-related personality scales Compared to the aggression alone group, the dual harm group scored significantly higher on psychopathy, aggression and hostility factors
Steeg et al. (57)	Denmark	Cohort of individuals born to Danish native parents, alive and residing in Denmark on their 15th birthday: 1: Those who had engaged in dual harm–145 2: Those who had engaged in self-harm alone–287 3: Those who had engaged in aggression alone–228	Hospital treated self-harm episodes since age of 10, irrespective of suicidal intent: identified from National Patient Register and the Psychiatric Central Research Register Violent crime since age of 15: identified from National Crime Register Dual harm: cross-tabulation of responses to the above measures	Substance misuse, psychiatric disorder: data from Psychiatric Central Research Register	Among those who died from any external cause, the prevalence of substance use disorders was higher in the dual harm group compared to the self-harm alone and aggression alone groups There was no significant differences in regard to other psychiatric disorders
Steinhoff et al. (68)	Switzerland	Sample of first-graders attending public school from the Zurich Project on Social Development from Childhood to Adulthood: 1. Those who had engaged in dual harm–107 2. Those who had engaged in self-harm alone–240 3. Those who had engaged in aggression alone–197	Self-harm, irrespective of suicidal intent: self-reported at ages 13, 15 and 17 using one item Aggression: response to an item from a broader delinquency scale, reported at 13, 15, and 17 Dual harm: cross-tabulation of responses to the above measures	Anxiety/depression at age 20: Social Behaviour Questionnaire Self-control at age 20: Self-Control Scale Psychopathy at age 20: The Short Dark Triad Substance use at age 20: 14 item questionnaire asking about past year substance use	Adjusted associations between behavioural groups at age 13–17 and psychological factors at age 20, controlling for sex, parental educational and migration background, and child’s education level at age 13: compared to no harm, self-harm alone, and aggression alone groups, dual harm group reported more anxiety/depression and psychopathy symptoms. Dual harm group also scored significantly higher on substance use and lack of self-control compared to no harm group.
Swogger et al. (69)	USA	Civil admission psychiatric patients: 1: Those who had engaged in dual harm–94 2: Those who had engaged in self-harm alone–149 3: Those who had engaged in aggression alone–144 4: Those who had engaged in no harmful behaviours–464	Self-harm, irrespective of suicidal intent: interview asking about self-harm behaviour during 10 weeks since the previous interview Aggression: follow-up interview and interviews with collateral informants. Not clear whether this was also assessed in the preceding 10 weeks Dual harm: cross-tabulation of responses to the above measures	Substance use disorder diagnosis: DSM-III-R checklist Psychopathy: PCL:SV Anger: Novaco Anger Scale	Unadjusted analysis: compared to the no harmful behaviours group, substance use disorder, anger and each psychopathy facet were significantly positively associated with dual harm Analysis adjusted for covariates: compared to no harmful behaviours group, anger and the antisocial facet of psychopathy predicted dual harm
Tardiff (60)	USA	Inpatients at psychiatric hospital: 1: Those who had engaged in dual harm–42 2: Those who had engaged in self-harm alone–52	Suicidal behaviour and physical aggression towards others in past 3 months: standardised measure reported by staff Dual harm: cross-tabulation of responses to the above measures	Psychopathology: adapted NOSIE scale	No significant differences in psychopathology
Watkins et al. (59)	USA	Veterans in residential treatment programme for post-traumatic stress disorder: 1: Those who had engaged in dual harm–202 2: Those who had engaged in no harmful behaviours–856 3: Those who had engaged in aggression alone–1,471 4: Those who had engaged in self-harm alone–41	Suicide attempt in past 4 months: one self-report question Aggression in past 4 months: self-report measure based on items in National Vietnam Readjustment Study Dual harm: cross-tabulation of responses to the above measures	Post-traumatic stress disorder symptoms: PTSD Checklist-Civilian for DSM-IV	More severe re-experiencing symptoms were related to a significantly higher probability of engaging in dual harm compared to no harmful behaviours Greater dysphoric arousal symptoms was related to a significantly higher probability of engaging in dual harm compared to no harmful behaviours Compared to dual harm, greater dysphoric arousal was significantly associated with a lower probability of engaging in self-harm alone Compared to dual harm, greater re-experiencing symptoms were significantly associated with a lower probability of engaging in aggression alone

#### 3.1.1. Non-homicide-suicide studies

Participants in the non-homicide-suicide studies were from community (*n* = 7), general psychiatric (including those discharged, *n* = 3), and forensic populations (*n* = 4 secure psychiatric; *n* = 2 prison). “Dual harm” was used to refer to the co-occurrence of self-harm and aggression in five studies (e.g., 54). Other terms used were “combined” or “co-occurring” aggression/violence (e.g., 55), and some did not use a specific term at all (e.g., 56). Most studies only assessed suicidal attempt (*n* = 9, e.g., 54), whilst six looked at self-harm irrespective of suicidal intent (e.g., 57). When examining aggression, six studies only assessed violent crime (e.g., 35). Five examined physical violence towards others (e.g., sexual assault, physical fights; 53), with Harford et al. (58) further examining stealing. Finally, four papers extended their definition of aggression by also assessing verbal aggression and property damage (e.g., 59).

More than half of the studies assessed harmful behaviours using bespoke non-validated self-report questionnaires that often comprised of one or two questions (*n* = 9, e.g., 55). Only two studies used validated questionnaires (31, 60) and others collected information through interviews (e.g., 56). Studies also obtained information from official records, such as psychiatric case files (e.g., 61), records of violent convictions and admissions to hospital (e.g., 57). Since there are no existing validated instruments for dual harm, this behaviour was examined by cross-tabulating responses to the separate self-harm and aggression measurements. Four studies used different timescales when assessing self-harm and aggression (e.g., 53), whilst three assessed lifetime history of both of these behaviours (52, 37, 56). The shortest time-period in which harmful behaviours were examined was 2 weeks prior to data collection (54). Three studies did not mention the time-period in which harmful behaviours were assessed (e.g., 62).

#### 3.1.2. Homicide-suicide studies

Seven studies examined a general homicide-suicide sample that was not defined by victim type. Other studies focussed on filicide (i.e., the killing of one’s child; *n* = 3) and intimate partner homicides (i.e., the killing of one’s intimate partner; *n* = 5). Most assessed completed suicide (*n* = 11, e.g., 63), whilst five examined attempted suicide (e.g., 64). Seven studies defined homicide-suicide as a suicidal act that occurred within 24 h after the homicide (e.g., 65). Similarly, Haines et al. (23) stated that the homicide had to have been perpetrated immediately before the suicide, but an exact timescale was not provided. Other studies did not restrict homicide-suicide to the above short timeline (e.g., 66) and five did not specify a timeline at all (e.g., 62). Homicide-suicide cases were identified from official case reports, such as those in official databases (e.g., National Violent Death Reporting System, government reports, death review committees; 65), files from forensic psychiatric settings (e.g., 67) and coroner reports (e.g., 23).

### 3.2. Risk of bias (quality) assessment

The risk of bias assessment for all studies is provided in Supplementary Appendix E. This assessment was agreed upon with an independent reviewer.

#### 3.2.1. Non-homicide-suicide studies

Most non-homicide-suicide studies were rated as having a moderate risk of bias (*n* = 10), followed by low (*n* = 5) and high risk of bias (*n* = 1). The majority of the research had used appropriate analytic methods (*n* = 15). Amongst the included papers, Richmond-Rakerd et al. (35), Steinhoff et al. (68), and Swogger et al. (69) adopted a longitudinal design with adequate follow-up periods (13 years, 3 years, 50 weeks, respectively). More than half of the studies were rated as being unbiased in the selection of their cohort (*n* = 10). However, there was not sufficient information to determine whether this criterion was met for five studies. Similarly, more than half of the included papers used a valid method for assessing predictor variables (e.g., validated questionnaires; *n* = 10). Other studies did not fully meet this criterion as they either failed to provide sufficient detail, utilised questionnaires that had not been validated, or relied on medical records without confirmation of the data by researchers. A common concern amongst the studies included lack of justification for their sample size. However, based on discussion with the research team, it was agreed that studies with sample sizes of more than 1,000 would have a lower risk of bias due to insufficient statistical power. Accordingly, seven other studies were rated as having met the criteria for having a justified sample size due to a large number of participants.

Half of the included research did not provide an adequate description of the different participant groups, adjust for pre-determined confounders or provide information on missing data (*n* = 8). Amongst the eight studies in which the researchers collected data, none reported blinding. Therefore, these studies may have been affected by researcher-related bias in which knowledge of how a participant scored on one measure may have influenced how the researcher scored other measures. A further frequent risk of bias was lack of valid method for ascertaining harmful behaviours (*n* = 12). For example, many studies used short self-report questionnaires that had not been validated. The greatest risk of bias was that no studies matched participant groups and so baseline differences in demographic factors between different groups were not minimised.

#### 3.2.2. Homicide-suicide studies

Similar to the non-homicide-suicide literature, most homicide-suicide studies were rated as having a moderate risk of bias (*n* = 6), closely followed by low (*n* = 5) and high risk of bias (*n* = 4). The use of appropriate analytical methods was the only criteria that was fully met by all studies. Another commonly met criteria was the use of valid methods to ascertain harmful behaviours (*n* = 14). The majority of studies utilised official databases, such as the National Violent Death Reporting System (NVDRS), that collate information from various sources. Such databases are often crosschecked to identify cases as accurately as possible and so may be considered to be a valid approach for assessing homicide-suicide, suicide alone and homicide alone cases. Most studies also met the criteria for having an unbiased selection of cohort. Again, this is due to the use of the above databases in most studies to identify eligible cases (*n* = 10). For example, the NVDRS, which was used by many of the included studies, reportedly holds the largest sample of homicide-suicide events amongst other existing datasets (65). Failing to control for pre-established confounders was a concern for almost half of the studies, which may have biased effect estimates (*n* = 7). Furthermore, the majority of studies did not minimise baseline differences in demographic factors between different groups of participants. Only Haines et al.’s (23) study met the above criteria by matching the homicide-suicide and suicide alone groups in age and sex. Furthermore, only five papers provided adequate descriptions of the different participant groups. Consequently, it was not possible to ascertain the extent to which individuals in the included research were representative of those with different demographic characteristics.

None of the included research provided a justification for their sample size. However, seven studies were rated as having met the criteria as they had a sample size over 1000. A common concern amongst the included papers was lack of valid method for assessing predictor variables (i.e., psychological factors). Almost half of the studies (*n* = 7) did not provide sufficient information to allow us to identify whether their assessment methods were valid. For example, in studies where mental health problems were identified from the NVDRS, it was often not specified whether this data was collected from a combination of sources, or from one source (e.g., only police reports). Since most studies analysed pre-existing data, being blind to participant status was not relevant to the majority of the research. This criterion only applied to one study, in which no blinding was reported (25). Finally, most studies did not report missing data and so we were unable to determine the extent of missing data and whether this was adequately handled (*n* = 13).

### 3.3. Are psychological factors uniquely associated with dual harm?

A summary of findings regarding differences in psychological factors between the behavioural groups (i.e., dual harm, self-harm alone, aggression alone, no harmful behaviours) is presented in Tables 2, 3. The trends identified from the included papers is demonstrated in Table 4. The psychological factors investigated in the papers included mental health problems and personality and emotion related factors. Below is the narrative synthesis of findings. Relevant statistics are provided where reported by studies.

**TABLE 4:** Summary of identified trends.

Psychological factor	Summary of trends
Mental health problems	Non-specific mental health problems: Significantly less likely to be linked to homicide-suicide when compared to suicide alone. Significantly more likely to be linked to homicide-suicide when compared to homicide alone.
	Mood disorder: Significantly less likely to be linked to homicide-suicide when compared to suicide alone. Significantly more likely to be linked to homicide-suicide when compared to homicide alone.
	Substance use disorder: Significantly more likely to be linked to less extreme forms of dual harm when compared to self-harm alone.
	Personality disorder: Significantly more likely to be linked to less extreme forms of dual harm when compared to self-harm alone.
Personality-related	Psychopathy (particularly impulsive/antisocial facet) significantly associated with less extreme forms of dual harm, but unclear whether this is uniquely associated with dual harm.
Emotion-related	Lack of sufficient evidence for how dual harm is associated with emotion-related factors.

#### 3.3.1. Mental health problems

Twenty-seven studies examined differences in various mental health problems between dual harm and other behavioural groups, including non-specific mental health problems, mood disorder (MD), anxiety disorder, psychotic disorders, personality disorders (PD), substance use disorder (SUD), and attention deficit hyperactivity disorder. Findings were largely mixed across studies, with no sufficient evidence that any of the above factors are uniquely associated with homicide-suicide or less extreme forms of dual harm. Nevertheless, there was some evidence that dual harm was significantly linked to non-specific mental health problems, MD, SUD, and PD when compared to *one* of the behavioural groups, but not when compared to *all* groups. This may suggest that these factors are not unique to dual harm, but rather driven by the separate self-harm or aggressive behaviours that constitute this act. Moreover, we found a different pattern of findings between homicide-suicide and non-homicide-suicide studies.

There was inconclusive evidence regarding how non-specific mental health problems and MD are linked to less extreme forms of dual harm. However, in regard to homicide-suicide, there was a trend for these factors to be significantly linked to this behaviour when compared to homicide alone [e.g., MD–(70), 37.3 vs. 20% for professionally diagnosed depression, 63.2 vs. 41.3% for non-professionally diagnosed depression; (62), 33 vs. 0%; (67), 31 vs. 7%; (25), 23 vs. 6%; mental health problems—(71), OR = 2.6; (21), OR = 4.4]. For example, Liem et al. (67) reported that when adjusting for variables such as gender, age, ethnicity and other psychiatric disorders, MD significantly raised the odds of a H-parasuicide (suicide attempt not resulting in death) by more than 15 times when compared to homicide alone. Moreover, Fridel and Zimmerman (65) found that the risk of a suicide after homicide increased by 341% for individuals with mental health problems. In contrast, when compared to suicide alone, there was evidence that those who had perpetrated homicide-suicide were significantly less likely to have a MD (21), [45 vs. 23%; (21), OR: 0.3; (66), OR = 0.3; (72), adjusted OR: 0.3; 63, adjusted OR = 0.5] and mental health problems [(21), OR: 0.5; (21), RRR = 0.1; (73), adjusted OR: 0.3; (72), adjusted OR: 0.4). This may suggest that the link found between these factors and homicide-suicide may be driven by the suicidal aspect of this behaviour. Benetiz-Borrego et al. (64), Heron (70), Vatnar et al. (63), Friedman et al. (74), and Liem et al. (67) further found that when age was stratified into those under (OR = 0.3) and over 30 years of age (OR = 0.3) in multivariate models adjusted for age, gender, ethnicity, marital status, and year of event, depression significantly decreased the risk of homicide-suicide when compared to those who had engaged in suicide alone. The above findings suggest that rather than be unique to homicide-suicide, MDs and mental health problems are linked to the suicidal behaviour of this act. It should be noted that several studies found no significant differences in these factors between homicide-suicide and other behavioural groups (72, 53, 62, 65, 74). Such differences in findings may be attributed to the high risk of bias present in the above research (e.g., unrepresentative sample) and the use of distinct methodologies (e.g., mental health problems assessed *via* professional mental health diagnoses vs. law enforcement reports).

Our review found evidence that SUDs and PDs may be linked to the aggressive behaviour in less extreme forms of dual harm, rather than be a unique characteristic of this behaviour. Harford et al. (53) examined differences in antisocial PD (ASPD), borderline PD (BPD), schizotypal PD, and DMS-5 SUDs between those who had engaged in dual harm, self-harm alone, aggression alone, and no harmful behaviours. When adjusting for sociodemographic characteristics and lifetime psychiatric disorders, the research found that alcohol, tobacco, and other drug use disorders had higher odds for dual harm [(53), OR = 1.4–1.7] when compared to self-harm alone, but not aggression alone. Similarly, ASPD was only significantly associated with dual harm when compared to no history of harmful behaviours (54, OR = 14.6) and self-harm alone (54, OR = 6.4). In support of the above findings, Harford et al. (75) revealed that every PD diagnostic criteria was significantly higher for dual harm only when this group was compared to self-harm alone (OR = 3.9). Moreover, three other studies found that those who had engaged in dual harm were significantly more likely to have SUDs when compared to individuals with a history of self-harm alone [(75), OR = 4.5, (35), OR = 3.3–4.3; (57), prevalence ratio = 1.8), but not aggression alone (37, 55, 68, 69).

Amongst homicide-suicide studies, whilst there was some empirical support for the notion that PDs may be associated with homicide-suicide when compared to self-harm alone (76, 67), this evidence was weak due to the small number of studies and their moderate to high risk of bias. In regard to SUD, only Benítez-Borrego et al.’s (64) study assessed this factor in relation to homicide-suicide and found no significant differences between those who had perpetrated filicide alone and filicide-suicide. Given that only one homicide-suicide study in this review examined SUD and this was rated as having a high risk of bias, there is not sufficient evidence for whether this factor is a distinguishing characteristic of extreme forms of dual harm. Nevertheless, in light of the trend that PD and SUD are significantly associated with less extreme forms of dual harm when compared to self-harm alone but not aggression alone, it may be that this relationship is driven by their link to the aggressive behaviour in dual harm.

#### 3.3.2. Personality related factors

No homicide-suicide studies assessed personality related factors in relation to harmful behaviours. Amongst non-homicide-suicide studies, four had examined differences in psychopathy between dual harm and other behavioural groups. Whilst findings suggested that this factor, in particular its impulsive and antisocial aspect, is significantly associated with dual harm, it was unclear whether this association is unique to dual harm or primarily driven by the separate self-harm or aggressive behaviours. Specifically, Stålenheim (38) found that compared to those who had engaged in repeated violent criminality alone, individuals who had engaged in dual harm were significantly more likely to score higher on personality factors representing Impulsive Sensation Seeking Psychopathy, Aggression and Hostility. However, no significant differences were found between those who had engaged in suicidal behaviour alone and dual harm (38). Similarly, Huang et al. (37) found that compared to the aggression alone group, those who had engaged in dual harm scored significantly higher on the anti-social subscale of the Psychopathy Checklist-revised. On the other hand, Swogger et al. (69) found that when adjusting for confounders, such as substance use, age, gender, and ethnicity, the antisocial facet of psychopathy was significantly associated with dual harm when compared to self-harm alone (OR = 1.6) and no harmful behaviours (OR = 1.6). Furthermore, Steinhoff et al. (68) found that when adjusting for sociodemographic factors, those who had engaged in dual harm reported significantly more psychopathy symptoms compared to the no harm (coefficient = 0.20), self-harm alone (coefficient = 0.13) and aggression alone (coefficient = 0.08) groups. Given the above mixed findings, there is insufficient evidence for whether psychopathy is uniquely associated with dual harm.

In regard to other personality related factors, Richmond-Rakerd et al. (35) examined whether the Big Five personality traits (i.e., extraversion, neuroticism, agreeableness, openness, and conscientiousness) are uniquely associated with dual harm. Depending on which behavioural group dual harm was compared to (i.e., self-harm alone, aggression alone or no harmful behaviours), extraversion, neuroticism, agreeableness, and openness showed a distinct pattern of associations with dual harm. This suggests that the above traits are not a unique aspect of dual harm, but may be driven by the risk associated with the separate self-harm and aggressive behaviours. However, lower conscientiousness (i.e., lower impulse control) was found to distinguish dual harm from all other behavioural groups, suggesting that lower impulse control may be a unique characteristic of this behaviour (e.g., self-harm alone vs. dual harm group, Cohen’s *d* = −0.6). Most of the above studies had a moderate risk of bias and so findings should be interpreted with caution.

#### 3.3.3. Emotion related factors

No homicide-suicide studies assessed emotion related factors in relation to harmful behaviours. Amongst non-homicide-suicide papers, two examined differences in anger amongst participants (69, 54). Swogger et al. (69) found that anger was significantly associated with dual harm amongst discharged psychiatric patients when compared to those without a history of harmful behaviours (OR = 1.02) and those with a history of self-harm alone, even when adjusting for confounders (e.g., sociodemographic characteristics). On the other hand, Hemming et al. (54) found no significant differences in anger between prisoners who had engaged in dual harm and both sole harm behaviours. Richmond-Rakerd et al.’s (35) found that compared to the self-harm alone group, those who had engaged in dual harm were significantly more likely to have low childhood self-control (OR = 1.8) and self-regulation difficulties as reported by caregivers (OR = 1.4) and teachers (OR = 1.6). In contrast, Steinhoff et al. (68) found that those who had engaged in dual harm were significantly more likely to have a lack of self-control only when compared to the no harm group (coefficient = 0.13), but not when compared to the self-harm alone or aggression alone groups. Given the above mixed findings, it is unclear how emotion related factors are associated with dual harm.

## 4. Discussion

This systematic review aimed to assess whether dual harm is distinguished by specific psychological factors when compared to self-harm alone, aggression alone and no harmful behaviours. The greatest commonality across the homicide-suicide and non-homicide-suicide literature is that findings are mixed. This is likely due to differences in methodologies and conceptualisations of harmful behaviours, as well as the moderate to high risk of bias present in most studies. Nevertheless, there is evidence that certain clinical factors, including MD, PD, SUD, and antisocial/impulsive related personality traits are associated with dual harm. There is a general trend for most studies to find differences in the above factors when comparing dual harm to only *one* of the behavioural groups (i.e., self-harm alone, aggression alone, or no harmful behaviours), but not when compared to *all* groups. Such findings suggest that these mechanisms are not uniquely associated with dual harm as a distinct clinical entity. Rather, they may be driven by the individual self-harm and aggressive behaviours that constitute dual harm. It is clear from our systematic review that further research is required in this field before a robust conclusion can be reached regarding the nature of dual harm.

### 4.1. Is dual harm a unique behavioural construct?

Our review found insufficient evidence that dual harm is associated with certain psychological factors when compared to *all* other behavioural groups. As such, findings do not support the hypothesis that dual harm is a unique behavioural construct with distinct characteristics. However, we found that some factors are associated with dual harm when this behaviour was compared to only *one* of the behavioural groups, but not the others (i.e., linked to self-harm alone but not aggression alone and vice versa). Such findings may suggest that the relationship found between certain psychological mechanisms and dual harm is driven by the individual self-harm or aggressive behaviour, rather than be associated with dual harm as a unique behavioural construct. For example, whilst homicide-suicide was significantly associated with mental health problems and MDs when compared to homicide alone, we found that these factors decreased the risk of homicide-suicide when compared to suicide alone. Accordingly, mental health problems and MDs are not unique to homicide-suicide, but may be linked to the suicidal aspect of this behaviour. In regard to PDs and SUDs, there was evidence that these factors are linked to the aggressive behaviour in less extreme forms of dual harm. Furthermore, there was evidence that impulsive and antisocial related traits (e.g., antisocial aspect of psychopathy, lower conscientiousness) are significantly associated with dual harm. However, due to mixed findings, it was unclear whether such factors are unique to dual harm, or driven by the separate risk associated with self-harm or aggression.

Our review found conflicting findings regarding mental health problems in homicide-suicide and non-homicide-suicide research. Such differences may reflect the distinct nature of these behaviours. For example, factors such as victim-offender relationship, intimate partner conflict and preceding stressors (e.g., marital conflict, financial problems), may be more likely to act as triggers for homicide-suicide than less extreme forms of dual harm (21, 66). It may be that rather than sit on the same continuum of behaviour, homicide-suicide is qualitatively distinct from less extreme forms of dual harm given its distinct conceptualisation and context. In order to assess whether it is meaningful to distinguish between homicide-suicide and non-homicide-suicide dual harm, studies could assess whether there are differences in how various psychological factors are associated with these behaviours. Furthermore, there is evidence that mental health problems are more prevalent amongst filicide-suicide perpetrators compared to intimate, family and extra-familial homicide-suicide (73). This could suggest that rather than approaching homicide-suicide perpetrators as a homogenous group, it is important to examine whether psychological differences exist between distinct types of homicide-suicide. Not distinguishing between subgroups of homicide-suicide perpetrators in our review may account for the lack of consistent findings regarding mental health problems in the included literature.

### 4.2. Theoretical support

Previous studies have demonstrated that dual harm is significantly associated with various factors when compared to other behavioural groups, leading to the hypothesis that this phenomena is a unique clinical construct with distinct characteristics. However, our review found insufficient evidence for the above notion. Findings suggest that rather than be linked to dual harm as a unique entity, the relationship found between dual harm and certain psychological factors may be driven by the separate self-harm and aggressive behaviours. Consequently, it may be more meaningful to consider dual harm as an overlap between self-harm and aggression and their risk factors, as opposed to a unique behavioural construct in its own right.

Plutchik and Van Praag’s (29) model of countervailing forces is in line with the above suggestion. The model suggests that the presence and interaction of factors that are separately associated with self-harm and aggression lead to an individual directing their underlying aggressive impulse towards both themselves and others (i.e., dual harm). Moreover, our findings support Boxer’s (4) notion that dual harm results from the presence of a wide range of risk factors that are associated with self-harm and aggression. Boxer (4) highlighted that from a developmental psychopathology stance, dual harm is an example of multifinality, in which a single range of risk factors can lead to different behavioural outcomes (i.e., self-harm or aggression). It may be that those who engage in both self-harm and aggression are likely to have experienced a “high loading” of risk across various factors linked to harmful behaviours (4, p. 206). It is the accumulation and multiplicative effect of such risk factors that may lead to the riskier profile demonstrated by those who dual harm.

Rather than accounting for dual harm through a unique framework, it may be more effective for theoretical models to consider how various risk factors associated with self-harm and aggression may interact to lead to dual harm. Drawing from existing models of harmful behaviours could provide a comprehensive account of how dual harm may emerge. For example, theories, such as Durkheim’s (19) social integration theory, that suggest homicide-suicide to be primarily driven by suicidal intent are supported by present findings that suggest mental health problems and MDs to be driven by the suicidal aspect of homicide-suicide. Nevertheless, it should be noted that Durkheim’s (19) theory focuses on the social mechanisms of this behaviour. Similarly, although the stream analogy of lethal violence (21) suggests that the psychological process of attribution underlies homicide-suicide, it is a primarily social framework that focuses on structural and cultural factors. Our review provides evidence for the link between psychological factors and dual harm (both homicide-suicide and less extreme forms). Additionally, previous research has identified various social and environmental factors that are associated with this behaviour (35–38). In light of such findings, it is important to adopt an interdisciplinary perspective that expands on existing theories and considers the myriad of psychological, social and environmental factors that may contribute to dual harm. For instance, Shafti et al.’s (15, 30) cognitive-emotional model adopts numerous existing theoretical frameworks, such as the general aggression model and diathesis-stress theories, in order to explain how various evidence-based risk factors of self-harm and aggression, including psychological factors, may interact to lead to dual harm. Findings from the present review that antisocial/impulsive related personality factors are associated with dual harm offer support to the cognitive emotional model of dual harm (15). The model proposes that a personality style, such as secondary psychopathy, may increase an individual’s risk of using both self-harm and aggression to regulate their negative emotions. However, the above model has not been empirically tested. It is important for future work to examine the psychological, social, and environmental factors that may contribute to dual harm in order to inform holistic theoretical accounts of this behaviour.

### 4.3. Critical appraisal

The secondary aim of this review was to conduct a critical appraisal of the included literature. Most homicide-suicide studies were rated as having an unbiased selection of cohort, largely due to the use of national databases. However, the studies were limited in their generalisability to non-Western countries. This is a concern given that cultural and structural differences across countries, such as cultural values, have been shown to influence harmful behaviours (77, 78). It is necessary to research dual harm in non-Western countries in order to assess differences in the aetiology of this behaviour across cultures. Many studies were limited in their design as they did not blind researchers to participant status or did not match different participant groups. The latter may have been of particular concern given that many papers found significant demographic differences between participant groups, including age, sex, and ethnicity. Moreover, the majority of the included research was cross-sectional. In order to provide stronger evidence for the causal role of psychological factors in dual harm, studies should assess the relationship between these variables over time using a longitudinal design.

There was variability in the definitions and measurements of harmful behaviours across the included research, reflected by the inconsistent terms used to refer to dual harm. A lack of agreed and empirically tested definition for dual harm is a major weakness of the literature, leading to variability in how this behaviour is conceptualised and assessed (30). For example, it has been debated whether it is clinically meaningful to consider a behaviour as dual harm if the self-harm and aggressive act co-occur at any point in time, or whether it is more appropriate to establish a restricted time-period (15). A priority in the literature should be to investigate the impact of adopting different definitions of dual harm. This may include restricting dual harm to different timeframes and severity of behaviours. Furthermore, whilst the standard definition of homicide-suicide is homicide immediately followed by suicide resulting in death of the perpetrator, some studies only assessed attempted suicide and did not restrict the timeline within which the two acts occurred. Future research should aim to use consistent measurements and conceptualisations of harmful behaviours to allow comparability. The importance of doing so is highlighted by reports that when broad definitions of harmful behaviours are adopted, the prevalence of dual harm is 3%, whilst narrower definitions provide a prevalence rate of 0.06% (53).

Many homicide-suicide studies did not use valid methods to measure mental health difficulties. Furthermore, in the case of suicide, it is challenging to measure psychological characteristics post-mortem, possibly leading to underreporting of such factors within the included research. A further concern is that self-reports of harmful behaviours have been found to be underreported and differ from medically and informant recorded data (79). Combing data across multiple sources (e.g., family reported, violent convictions, self-report, hospital admissions) may help in future research.

Most studies did not carry out a power analysis and so a lack of significant findings in papers with small sample sizes may have been attributed to inadequate power. Furthermore, half of the included research did not account for pre-determined confounders. This is a concern as various environmental, sociodemographic and psychological factors have been found to be associated with harmful behaviours. For example, Harford et al.’s (75) paper found that the likelihood of having experienced physical and sexual abuse was significantly higher in those who had engaged in dual harm (OR = 2.7, OR = 2.8, respectively) when compared to aggression alone. This relationship was modified by psychiatric disorders and sociodemographic factors. Given that we found various psychological factors to be associated with dual harm, future research should adopt multilevel theorising and multivariate analyses in order to capture the complexity of how various mechanisms may interact to lead to co-occurring self-harm and aggression.

### 4.4. Implications

At this stage, given the limitations within the literature, it is premature to recommend whether or not dual harm should be established as a unique behavioural construct within clinical practice. Nevertheless, this review adds to the growing literature by extending our understanding of the characteristics of dual harm and the nature of their relationship to this behaviour. Our findings highlight that approaching self-harm and aggression separately within research and practice may be insufficient and that it is imperative to consider the potential duality of an individual’s harmful behaviours (15). For example, it may be important to identify risk factors of self-harm in those who have engaged in aggression and vice versa, in order to lessen the likelihood of their co-occurrence. Identifying the extent to which an individual has been exposed to such factors, as well as careful consideration of their history of other harmful behaviours, may aid the identification of those who are likely to engage in future dual harm. Furthermore, a transdiagnostic approach that identifies the common underlying mechanisms of factors associated with self-harm and aggression and aims to reduce an individual’s level of risk across such factors may help to prevent dual harm.

The present work highlighted the association between dual harm and various mental health (e.g., PD, SUD, MD) and personality related factors (e.g., antisocial facet of psychopathy, impulsivity) linked to self-harm and aggression. Future research should build upon this review by further investigating the link between these mechanisms and dual harm amongst forensic populations. Stronger evidence for the role of such psychological factors in dual harm would demonstrate the importance of their identification and treatment in risk assessments and interventions of harmful behaviours within forensic settings.

Whilst dual harm may result from the presence of risk factors that are separately associated with self-harm and aggression, it may be that these behaviours are used interchangeably to serve the same purpose in individuals who dual harm (e.g., regulating negative emotions; 15). Therefore, rather than approach self-harm and aggression separately, a key consideration for clinicians and future research may be to assess whether these behaviours are used to fulfil a shared function in the context of dual harm. Furthermore, although it may not be clinically meaningful to approach dual harm as an independent behavioural construct, it is important to recognise the distinct needs and risk profile shown by those who engage in this behaviour. For example, there should be a recognition of barriers to treatment that may be unique to those who engage in both self-harm and aggression as a result of the duality of their harmful behaviours and greater level of risk (15, 57). Steeg et al. (57) further highlighted that those who engage in dual harm are likely to have been in contact with healthcare, criminal justice, and social services. Therefore, a coordinated effort from the above sectors may allow more effective risk-assessment, prevention and treatment strategies for these individuals.

In regard to homicide-suicide, the most common finding was that those with a history of suicide alone are more likely to have a mood disorder and mental health problems compared to homicide-suicide. In a study of violent and non-violent patients, Apter et al. (80) found distinct patterns of correlations between various factors and the risk of suicide. Whilst in the violent group there was a significant correlation between anger and suicide risk (*r* = 0.7), there was a significant relationship between sadness and suicidal risk in non-violent patients (*r* = 0.5). Furthermore, happiness was negatively associated with suicidal risk in the non-violent participants (*r* = −0.6). Alongside the findings of the present review, it may be plausible to suggest that suicidal behaviour alone and suicidal behaviour in those who dual harm may have different underlying mechanisms. As such, it may be that distinct approaches should be used to manage suicide risk in those who have also engaged in extreme forms of aggression and those who have not. Nevertheless, given conflicting findings within the homicide-suicide literature and the limitations of such research, there is a need for future investigations of the aetiology of homicide-suicide that provide stronger evidence-based implications.

### 4.5. Limitations and strengths

This review should be understood in light of its limitations. The included studies were limited to those published in English and those that had examined adults. Therefore, we may have failed to identify relevant non-English papers and findings may not generalise to younger populations. It is important for future research to examine dual harm amongst younger samples in order to inform our understanding of the development and aetiology of this behaviour. Furthermore, self-harm was assessed more generally by not distinguishing between suicidal and non-suicidal forms of self-harm. Finally, it was not possible to conduct a meta-analysis and compute an absolute effect regarding how psychological factors are associated with dual harm. Therefore, this work should be considered as an exploratory systematic review that provides preliminary evidence for the nature of dual harm.

Despite its limitations, to the best of our knowledge, this work is the first systematic review to investigate differences in psychological characteristics between those who have engaged in dual harm, sole harm behaviours and no harmful behaviours. Integrating findings has allowed us to provide important contributions to the emerging field of dual harm by critically reviewing literature in light of previous theories and identifying gaps to be addressed by future research. Additionally, this review followed best practice by adopting PRISMA (39) and Economic and Social Research Council guidelines (51). Finally, by having an independent reviewer conduct checks at each stage of the review, we have reduced the risk of bias. This is evident by the almost perfect agreement between the lead and independent reviewer in the screening.

## 5. Conclusion

A holistic view of the literature provides preliminary evidence that psychological factors that at first glance seem to be uniquely associated with dual harm, are actually likely to be driven by their association with the separate self-harm or aggressive behaviours. These findings suggest that dual harm is not a unique clinical entity. Rather, it is the complex interactions between risk factors associated with self-harm and aggression and their multiplicative effect that may lead to dual harm. Whilst there has historically been a separation in how we perceive and approach self-harm and aggression, our review highlights the importance of adopting an integrated approach that assesses these behaviours and their risk factors together in the context of dual harm. Doing so may aid the prevention and management of co-occurring self-harm and aggression within forensic and clinical settings. Furthermore, our critical appraisal identified areas of improvement for future research. Studies that follow the recommendations provided by this review will help extend our understanding of those who engage in dual harm, and thereby provide important implications for clinical practice.

## Data availability statement

The original contributions presented in this study are included in this article/Supplementary material, further inquiries can be directed to the corresponding author.

## Author contributions

MS, AF, DP, and PT were involved in the conceptualisation, design, and planning of the review. MS was involved in writing the protocol, literature searches, risk of bias assessment, data extraction, synthesis, and writing the manuscript. MS and FH were involved in the screening. AF, DP, FH, and PT contributed to reading and revision of the manuscript. All authors contributed to the article and approved the submitted version.
