# Supplementary material for: A systematic review of the co-occurrence of self-harm and aggression: Is dual harm a unique behavioural construct?
Source: Front Psychiatry. 2023 Feb 16;14:1083271. doi: 10.3389/fpsyt.2023.1083271 (PMC9978485; doi:10.3389/fpsyt.2023.1083271)
Supplement: Supplementary file 1 [file Table_1.docx]

**Supplementary Material**

Appendix A. PRISMA Checklist

| **Section and Topic** | **Item #** | **Checklist item** | **Location where item is reported** |
| --- | --- | --- | --- |
| **TITLE** | | |  |
| Title | 1 | Identify the report as a systematic review. | Page 1 |
| **ABSTRACT** | | |  |
| Abstract | 2 | See the PRISMA 2020 for Abstracts checklist. | Page 1 |
| **INTRODUCTION** | | |  |
| Rationale | 3 | Describe the rationale for the review in the context of existing knowledge. | Page 1-4 |
| Objectives | 4 | Provide an explicit statement of the objective(s) or question(s) the review addresses. | Page 3 |
| **METHODS** | | |  |
| Eligibility criteria | 5 | Specify the inclusion and exclusion criteria for the review and how studies were grouped for the syntheses. | Page 4-6 |
| Information sources | 6 | Specify all databases, registers, websites, organisations, reference lists and other sources searched or consulted to identify studies. Specify the date when each source was last searched or consulted. | Page 4 |
| Search strategy | 7 | Present the full search strategies for all databases, registers and websites, including any filters and limits used. | Page 4 |
| Selection process | 8 | Specify the methods used to decide whether a study met the inclusion criteria of the review, including how many reviewers screened each record and each report retrieved, whether they worked independently, and if applicable, details of automation tools used in the process. | Page 4-5 |
| Data collection process | 9 | Specify the methods used to collect data from reports, including how many reviewers collected data from each report, whether they worked independently, any processes for obtaining or confirming data from study investigators, and if applicable, details of automation tools used in the process. | Page 5 |
| Data items | 10a | List and define all outcomes for which data were sought. Specify whether all results that were compatible with each outcome domain in each study were sought (e.g. for all measures, time points, analyses), and if not, the methods used to decide which results to collect. | Page 5 |
|  | 10b | List and define all other variables for which data were sought (e.g. participant and intervention characteristics, funding sources). Describe any assumptions made about any missing or unclear information. | Page 5 |
| Study risk of bias assessment | 11 | Specify the methods used to assess risk of bias in the included studies, including details of the tool(s) used, how many reviewers assessed each study and whether they worked independently, and if applicable, details of automation tools used in the process. | Page 5 |
| Effect measures | 12 | Specify for each outcome the effect measure(s) (e.g. risk ratio, mean difference) used in the synthesis or presentation of results. | Page 5-6 |
| Synthesis methods | 13a | Describe the processes used to decide which studies were eligible for each synthesis (e.g. tabulating the study intervention characteristics and comparing against the planned groups for each synthesis (item #5)). | Page 4-6 |
|  | 13b | Describe any methods required to prepare the data for presentation or synthesis, such as handling of missing summary statistics, or data conversions. | Page 5-6 |
|  | 13c | Describe any methods used to tabulate or visually display results of individual studies and syntheses. | Page 6 |
|  | 13d | Describe any methods used to synthesize results and provide a rationale for the choice(s). If meta-analysis was performed, describe the model(s), method(s) to identify the presence and extent of statistical heterogeneity, and software package(s) used. | Page 5-6 |
|  | 13e | Describe any methods used to explore possible causes of heterogeneity among study results (e.g. subgroup analysis, meta-regression). | N/A |
|  | 13f | Describe any sensitivity analyses conducted to assess robustness of the synthesized results. | N/A |
| Reporting bias assessment | 14 | Describe any methods used to assess risk of bias due to missing results in a synthesis (arising from reporting biases). | Page 5 |
| Certainty assessment | 15 | Describe any methods used to assess certainty (or confidence) in the body of evidence for an outcome. | Page 5 |
| **RESULTS** | | |  |
| Study selection | 16a | Describe the results of the search and selection process, from the number of records identified in the search to the number of studies included in the review, ideally using a flow diagram. | Page 7 |
|  | 16b | Cite studies that might appear to meet the inclusion criteria, but which were excluded, and explain why they were excluded. | Page 6 |
| Study characteristics | 17 | Cite each included study and present its characteristics. | Table 2 and 3 |
| Risk of bias in studies | 18 | Present assessments of risk of bias for each included study. | Appendix E |
| Results of individual studies | 19 | For all outcomes, present, for each study: (a) summary statistics for each group (where appropriate) and (b) an effect estimate and its precision (e.g. confidence/credible interval), ideally using structured tables or plots. | Table 2 and 3 |
| Results of syntheses | 20a | For each synthesis, briefly summarise the characteristics and risk of bias among contributing studies. | Page 6-14, Table 2 and 3 |
|  | 20b | Present results of all statistical syntheses conducted. If meta-analysis was done, present for each the summary estimate and its precision (e.g. confidence/credible interval) and measures of statistical heterogeneity. If comparing groups, describe the direction of the effect. | N/A |
|  | 20c | Present results of all investigations of possible causes of heterogeneity among study results. | N/A |
|  | 20d | Present results of all sensitivity analyses conducted to assess the robustness of the synthesized results. | N/A |
| Reporting biases | 21 | Present assessments of risk of bias due to missing results (arising from reporting biases) for each synthesis assessed. | Page 6-14 |
| Certainty of evidence | 22 | Present assessments of certainty (or confidence) in the body of evidence for each outcome assessed. | Page 6-14 |
| **DISCUSSION** | | |  |
| Discussion | 23a | Provide a general interpretation of the results in the context of other evidence. | Page 16-18 |
|  | 23b | Discuss any limitations of the evidence included in the review. | Page 17 |
|  | 23c | Discuss any limitations of the review processes used. | Page 18 |
|  | 23d | Discuss implications of the results for practice, policy, and future research. | Page 17-18 |
| **OTHER INFORMATION** | | |  |
| Registration and protocol | 24a | Provide registration information for the review, including register name and registration number, or state that the review was not registered. | Page 4 |
|  | 24b | Indicate where the review protocol can be accessed, or state that a protocol was not prepared. | Page 4 |
|  | 24c | Describe and explain any amendments to information provided at registration or in the protocol. | Page 4 |
| Support | 25 | Describe sources of financial or non-financial support for the review, and the role of the funders or sponsors in the review. | Page 19 |
| Competing interests | 26 | Declare any competing interests of review authors. | Page 19 |
| Availability of data, code and other materials | 27 | Report which of the following are publicly available and where they can be found: template data collection forms; data extracted from included studies; data used for all analyses; analytic code; any other materials used in the review. | Page 19 |

Appendix B. Characteristics of included studies

| **Reference** | **Country** | **Study design** | **Relevant groups of study - N of participants** | **Age (mean, SD)** | **Female (%) in each group** | **Harmful behaviours examined and measures** | **Psychological factor(s) examined: measure(s)** | **Reported findings** |
| --- | --- | --- | --- | --- | --- | --- | --- | --- |
| Benítez-Borrego, Guàrdia-Olmos & Aliaga-Moore (2013) | Chile | Cross-sectional | 1: Filicide-suicide perpetrators - 33  2: Filicide alone perpetrators - 35 | Filicide-suicide: 34.76(9.68)  Filicide alone: 32.77(11.4) | Filicide-suicide: 64  Filicide alone: 49 | Filicide-suicide (attempted suicide within 24 hours of filicide) and filicide alone: cases reported in the Legal Medical Service | Diagnosed psychiatric symptoms: forensic reports from forensic psychiatric and psychological evaluations in Legal Medical Service | No significant differences in diagnosed psychiatric symptoms |
| Flynn, Swinson, While, Hunt, Roscoe, Rodway, Windfuhr, Kapur, Appleby, & Shaw (2009) | England & Wales | Cross-sectional | 1: H-S perpetrators - 203  2: Those who had engaged in suicide alone – 46,358  3: Homicide alone perpetrators – 5,096 | Only median(range) reported:    H-S: 44(18-88)  Suicide alone: 42(10-102)  Homicide alone: 28(9-99) | H-S: 14  Suicide alone: 25  Homicide alone: 10 | Homicide alone, completed suicide and H-S (completed suicide, 3 groups of timescales: suicide immediately after homicide, suicide more than 3 days after homicide but before conviction, and suicide after conviction): case records from Home Office and police | Mental health problems: questionnaire completed by individual’s mental health team | Compared to H-S perpetrators, those who engaged in suicide alone were significantly more likely to have a severe mental illness, while H-S perpetrators were significantly more likely to have a personality disorder  No significant differences between homicide alone and H-S groups in schizophrenia, affective disorder and personality disorder |
| Fridel & Zimmerman (2019a) | USA | Cross-sectional | 1: H-S perpetrators – 2,048    2: Those who had engaged in suicide alone – 103,195 | H-S: 45.7(16.3)  Suicide alone: 46.9(17.1) | H-S: 7  Suicide alone: 22 | Suicide alone and H-S (completed suicide within 24 hours of homicide): cases of deaths reported by the NVDRS in 2003 – 2013 | Mental health stressors: coroner/medical examiner reports and law enforcement reports in the NVDRS | All mental health stressors were significantly more prevalent amongst those who had engaged in suicide alone than H-S perpetrators |
| Fridel & Zimmerman (2019b) | USA | Cross-sectional | 1: H-S perpetrators – 1,413    2: Homicide alone perpetrators – 22,960  3: Those who had engaged in suicide alone – 81,179 | H-S: 45(16.39)  Homicide alone: 39.95(24.99)  Suicide alone: 46.62(17.25) | H-S: 7  Homicide alone: 9  Suicide alone: 22 | H-S (completed suicide within 24 hours of homicide), homicide alone, suicide alone: cases of deaths reported by the NVDRS in 2003 - 2013 | Mental health: coroner/medical examiner records and law enforcement reports in the NVDRS | H-S perpetrators were significantly more likely to have mental health problems than homicide alone perpetrators, with the risk of a suicide after homicide increasing for those with mental health problems  Those who engaged in suicide alone were significantly more likely to have mental health problems than H-S perpetrators |
| Friedman, Holden, Hrouda, & Resnick (2008) | USA | Cross-sectional | 1: Mothers who had perpetrated filicide followed by non-fatal and fatal suicide - 29  2: Mothers who had perpetrated filicide alone - 20 | Filicide-completed suicide: 31.8(5.7)  Filicide-attempted suicide: 29.9(6.3)  Filicide alone:  28(5.7) | 100 | Filicide-suicide and filicide alone: records from coroner's office | Psychotic symptoms and depression: not specified | Compared to filicide alone and filicide-attempted suicide, mothers who engaged in filicide-completed suicide were significantly less likely to have been noted to be delusional  There were no significant differences in depression, auditory hallucinations or command auditory hallucinations |
| Haines, Williams & Lester (2010) | Tasmania | Cross-sectional | 1: H-S perpetrators – 22    2: Those who had engaged in suicide alone - 22 | Only reported for entire sample: 39.2(12.7) | 0 | Suicide and H-S (completed suicide immediately after homicide): files from coroners at the Tasmanian Archives Office and Department of Justice | Psychological symptoms: files from coroners | There was a trend for fewer of the H-S group to have experienced anxiety in the time leading up to their death. |
| Heron (2017) | Canada | Cross-sectional | 1: Intimate H-S perpetrators – 64  2: Intimate homicide alone perpetrators - 158 | Intimate H-S: 48.13  Intimate homicide alone: 39.11 | 0 | Intimate H-S (completed suicide, no timescale specified) and intimate homicide alone: case records from the Ontario DVDRC | Depression and other mental health problems: records from Ontario DVDRC. Diagnosis of depression based on the opinion of professionals and non-professionals | There was a significantly larger amount of H-S perpetrators who had been professionally and unprofessionally diagnosed with depression than homicide alone perpetrators  H-S and homicide alone perpetrators did not significantly differ based on other psychiatric diagnoses |
| Kalesan, Mobily, Vasan, Siegel, & Galea (2018) | USA | Cross-sectional | 1: H-S perpetrators – 1,422    2: Those who had engaged in suicide alone- 41,244 | Not reported | H-S: 7  Suicide alone: 14 | H-S (homicide occurred in the preceding year to the completed suicide) and suicide alone: cases of deaths reported by the NVDRS in 2003 - 2011 | Depression and mental health issues: NVDRS records | Across all ages, depression decreased risk of H-S with a firearm compared to suicide alone  Depression and mental health issues decreased risk of H-S compared to suicide alone in those under 30 years old and over 30 years old |
| Leveillee, Marleau & Dube (2007) | Canada | Cross-sectional | 1: Filicide-suicide perpetrators - 38  2: Filicide alone perpetrators - 37 | Filicide-suicide: 45  Filicide alone: 59 | Not reported | Filicide-suicide (outcome of suicidal behaviour and timescale not specified) and filicide alone: files of cases compiled by the Bureu du Coroner en Chef du Quebec | Depressive and psychotic disorders: coroner’s reports, psychiatric and medical records, summaries of investigations by the Youth Protection Commission and the Youth Protection Directorate | Males who engaged in filicide-suicide were significantly more likely than those who engaged in filicide alone to have depressive disorders |
| Liem, Hengeveld & Koenraadt (2009) | Netherlands | Cross-sectional | 1: Homicide-parasuicide perpetrators - 77  2: Homicide alone perpetrators - 430  3: Those who had engaged in parasuicide alone - 160 | Homicide-parasuicide: 37  Homicide alone: 33.1  Parasuicide alone: 38.3 | Homicide-parasuicide: 22  Homicide alone: 24  Parasuicide alone: 27 | Homicide-parasuicide (near lethal suicidal behaviour, timescale not specified) and homicide alone: cases reported in a forensic psychiatric hospital    Parasuicide alone (near lethal suicidal behaviour) – cases reported in a psychiatric hospital. Classified as a parasuicide using the Pierce Suicide Intent Scale | Psychopathological characteristic based on DSM-IV: case files in psychiatric hospital. If diagnosis not in files, a retrospective diagnosis was made according to file information | Homicide–parasuicide perpetrators were significantly more likely than homicide alone perpetrators to have a mood disorder, most notably depression.  Depression raised the odds of a parasuicide following a homicide more than 15 times. However, there were no differences in psychotic disorder or personality disorder.  Homicide-parasuicide perpetrators were significantly more likely to be diagnosed with a psychotic disorder and personality disorder compared to the parasuicide alone group. However, there were no significant differences in mood disorders |
| Liem, & Roberts (2009) | Netherlands | Cross-sectional | 1: Intimate H-S perpetrators – 44  2: Intimate homicide alone perpetrators - 297 | Intimate H-S: 38.8(13.4)  Intimate homicide alone: 36.5(10.5) | 0 | Intimate H-S (suicidal behaviour near lethal, timescale not specified) and intimate homicide alone: archive of clinical records in a forensic psychiatric hospital | Psychopathology: case records from forensic psychiatric hospital | The H-S and homicide alone groups did not significantly differ in psychotic disorders  H-S perpetrators were significantly more likely to be diagnosed with a depressive disorder |
| Logan, Hill, Black, Crosby, Karch, Barnes, & Lubell (2008) | USA | Cross-sectional | 1: H-S perpetrators – 408    2: Those who had engaged in suicide alone – 20,183 | Only median for H-S perpetrators reported: 43 | H-S: 9  Suicide alone: 22 | H-S (completed suicide 24 hours after homicide) and suicide alone: cases of deaths reported by the NVDRS in 2003-2005 | Mental health problems, depressed mood: NVDRS records | Compared with males who engaged in suicide alone, male H-S perpetrators were significantly less likely to have reports of depressed mood and mental health problems |
| Logan, Ertl & Bossarte (2019) | USA | Cross-sectional | 1: Intimate H-S perpetrators – 1,504  2: Those who had engaged in suicide alone – 28,755 | Intimate H-S perpetrators: 46.23(15.6)  Suicide alone: 40.47(13.7) | 0 | Intimate H-S (completed suicide within 24 hours of homicide) and completed suicide alone: cases of deaths reported by the NVDRS in 2003 - 2015 | Current depressed mood and mental health condition: NVDRS records | Intimate homicide perpetration was less prevalent among suicide decedents who had a known current depressed mood and mental health conditions |
| Vatnar, Friestad, & Bjørkly (2019) | Norway | Cross-sectional | 1: Intimate H-S perpetrators - 44  2: Intimate homicide alone perpetrators - 133 | Intimate H-S: 44.9  Intimate homicide alone: 38.5 | Intimate H-S: 2  Intimate homicide alone: 14 | Intimate Homicide alone and H-S (suicide within 24 hours of homicide): cases identified from the NCIS | Professionally diagnosed mental health diagnosis: reports from NCIS statistics | No significant differences in professionally diagnosed mental health diagnosis |
| Zimmerman & Fridel (2020) | USA | Cross-sectional | 1: H-S perpetrators – 2,535  2: Homicide alone perpetrators – 28,027  3: Those who had engaged in suicide alone – 138,948 | H-S: 45.81(16.3)  Homicide alone: 32.41(13.59)  Suicide alone: 46.13(17.85) | H-S: 7  Homicide alone: 10  Suicide alone: 22 | H-S (completed suicide within 24 hours of homicide), homicide alone, and completed suicide: cases of deaths reported by the NVDRS in 2003 - 2015 | Mental health problems: NVDRS records | Odds of suicide following homicide were significantly elevated for perpetrators with mental health problems |
| Ghossoub, Adib, Maalouf, Fuleihan, Tamim, & Nahas (2019) | USA | Cross-sectional | Nationally representative sample of noninstitutionalised , household-based civilian population from NSDUH:  1: Those who had engaged in dual harm - 410  2: Those who had engaged in no harmful behaviours – 259,914 | No information on mean(SD) provided.  Minimum age 18 for all participants. | Dual harm: 54  No history of harmful behaviours: 52 | Past year suicidal behaviour and physical attacks towards others: based on self-reported answers to two questions  Dual harm: cross-tabulation of responses to the above measures | Past year SUD and psychiatric disorder: self-reported survey based on DSM-IV criteria | Compared to those who had no history of harmful behaviours, substance use disorders and psychiatric disorders were significantly more prevalent in the dual harm group  Alcohol use disorders, drug use disorders and alcohol and drug use disorders significantly increased the odds of perpetrating dual harm compared to having no history of harmful behaviours, even after adjusting for sociodemographic characteristics |
| Harford, Yi & Grant (2014) | USA | Cross-sectional | Civilian noninstitutionalised population from NESARC  1: Those who had engaged in dual harm - 688  2: Those who had engaged in aggression alone – 4,689  3: Those who had engaged in self-harm alone - 996 | No information on mean(SD) provided.  Minimum age 18 for all participants. | Dual harm: 67  Aggression alone: 43  Self-harm alone: 73 | Lifetime physical aggression towards others: bespoke 5 item self-report questionnaire  Suicidal behaviour: based on one question asking about lifetime suicidal attempt and one question asking about suicidal behaviour in those who screened positive for a DSM-IV major depressive episode.  Dual harm: cross-tabulation of responses to the above measures | Lifetime DSM-IV diagnosis of psychiatric disorders: Alcohol Use Disorder and Associated Disabilities Interview Schedule | Odds of substance use disorder was significantly higher for dual harm group compared to self-harm alone group  Odds of personality disorder was significantly higher for dual harm group compared to self-harm alone and aggression alone group  Odds of mood disorders was significantly higher for dual harm group compared to self-harm alone and aggression alone group  Odds of anxiety disorders was significantly higher for dual harm group compared to aggression alone group |
| Harford, Chen, Kerridge, & Grant (2018a) | USA | Cross-sectional | Civilian noninstitutionalised population from NESARC-III:  1: Those who had engaged in dual harm – 1,060  2: Those who had engaged in aggression alone – 4,038  3: Those who had engaged in self-harm alone – 1,730  4: Those who had engaged in no harmful behaviours – 29,481 | Dual harm: 39.6  Aggression alone: 43.3  Self-harm alone: 42.9  No harmful behaviours: 47.4 | Dual harm: 57  Aggression alone: 32  Self-harm alone: 70  No harmful behaviours: 53 | Suicidal behaviour: based on one question asking about lifetime suicidal attempt and one question asking about suicidal behaviour in preceding two weeks during the time they experienced depression or mania  Aggression: had engaged in at least one of 7 aggressive behaviours since age of 15. Not specified whether these questions were self-reported. Behaviours included stealing from someone, sexual assaults, fights, physical aggression towards others, using a weapon in a fight  Dual harm: cross-tabulation of responses to the above measures | Lifetime DSM-IV diagnosis of psychiatric disorders: Alcohol Use Disorder and Associated Disabilities Interview Schedule | Substance use disorder, bipolar 1 disorder, panic disorder, generalized anxiety disorder, post-traumatic stress disorder, schizotypal personality disorder, antisocial personality disorder, and borderline personality disorder were significantly associated with higher odds for dual harm relative to aggression alone and self-harm alone  When adjusting for sociodemographic characteristics and lifetime DSM-5 disorders -  all substance use disorders showed significantly higher odds for dual harm relative to no history of harmful behaviours.  Alcohol, tobacco, and other drug use disorders showed significantly higher odds for dual harm relative to self-harm alone.  Mood disorders showed significantly higher odds for dual harm relative to history of no harmful behaviours and aggression alone. Post-traumatic stress disorder, schizotypal personality disorder, antisocial personality disorder and borderline personality disorder showed significantly higher odds for dual harm relative to no history of harmful behaviours. Antisocial personality disorder and borderline personality disorder also had significantly higher odds for dual harm relative to self-harm alone, as did borderline personality disorder for dual harm relative to aggression alone |
| Harford, Yi, Chen, & Grant (2018b) | USA | Cross-sectional | Nationally representative sample of noninstitutionalised , household-based civilian population from NSDUH:  1: Those who had engaged in dual harm – 464  2: Those who had engaged in self-harm alone – 2,289  3: Those who had engaged in aggression alone - 7,286  4: Those who had engaged in no harmful behaviours - 304,842 | No information on mean(SD) provided.  Minimum age 18 for all participants | Dual harm: 54  Aggression alone: 38.1  Self-harm alone: 58.4  No harmful behaviours: 51.9 | Past year suicidal behaviour and physical attacks towards others: based on self-reported answers to two questions  Dual harm: cross-tabulation of responses to the above measures | Substance use disorders: based on DSM-IV diagnoses, but not reported how this was assessed  Nicotine dependence: Nicotine Dependence Syndrome Scale and the Fagerstrom Test of Nicotine Dependence  Serious psychological distress: Kessler-6 | Compared with the self-harm alone, aggression alone and no harmful behaviours groups, the dual harm group were significantly more likely to have serious psychological distress, nicotine dependence and four or more DSM-IV SUD criteria for alcohol, cocaine, pain reliever, and stimulant use disorders  Compared to self-harm alone and no harmful behaviours groups, the dual harm group was significantly more likely to have four or more DSM-IV marijuana use disorder criteria |
| Hemming, Shaw, Haddock, Carter, & Pratt (2021) | UK | Cross-sectional | 1: Prisoners who had engaged in dual harm – 12  2: Prisoners who had engaged in self-harm alone – 4  3: Prisoners who had engaged in aggression alone - 25 | Only total sample median and range reported  Median = 32 Range = 21–56 | 0 | Aggression assessed over past 2 weeks: bespoke 5 item questionnaire assessing physical aggression, property damage, verbal aggression  Suicide alone assessed over past 2 weeks: bespoke 6 item questionnaire    Dual harm: cross-tabulation of responses to the above measures | Alexithymia: Toronto Alexithymia Scale  Anger: the Novaco Anger Scale  Impulsivity: the Dickman Impulsivity Inventory | No significant differences in alexithymia, anger or impulsivity |
| Hillbrand (1992) | Not reported (assumed to be USA) | Cross-sectional | Forensic psychiatric patients with a history of severe violence:  1: Those who had engaged in aggression alone - 35  2: Those who had engaged in dual harm - 15 | Dual harm: 30.9(7.6)  Aggression alone: 28.6(6.2) |  | Self-harm irrespective of suicidal intent and aggression (physical, verbal, property damage) during a 6 month period: Overt aggression scale  Dual harm: cross-tabulation of responses to the above measures | Psychiatric diagnosis: medical records | No significant differences in  in personality disorders, alcohol/substance abuse or psychotic disorders |
| Huang, Zhang, Zhong, Gou, Sun, Guo, Lin, Guo, Chen, Wang, Zhou, & Wang, 2022 | China | Cross-sectional case-control | Individuals with serious aggressive behaviours and suspected mental disorder in seven forensic institutes in different provinces  1. Those who had engaged in dual harm – 74  2. Those who had engaged in aggression alone – 349 | Dual harm: 33.53(9.29)  Aggression alone: 34.40(10) | Dual harm: 16.2  Aggression alone: 8 | Lifetime self-harm (unclear if non-suicidal self-harm assessed): self-report questionnaire  Serious aggressive behaviours: participants’ forensic archives  Dual harm: cross-tabulation of responses to the above measures | History of substance abuse and mental disorders: standardised data collection form and forensic archives  Current mental disorder: evaluated by two psychiatrists using ICD-10  Psychopathy: Chinese version of Psychopathy Checklist-Revised (PCL-R)  Psychiatric symptoms: Chinese version of Brief Psychiatric Rating Scale (BPRS) | Compared to the aggression alone group, the dual harm group were significantly more likely to have a history of mental disorder, current mental disorder, score higher on the anti-social scale of the PCL-R, and score higher on the anxiety-depression scale of the BPRD. There were no significant differences in substance use. |
| Laporte, Ozolins, Westling, Westrin, Billstedt, Hofvander, & Wallinius (2017) | Sweden | Cross-sectional | Young adult violent offenders:  1: Those who had engaged in dual harm – 62  2: Those who had engaged in aggression alone - 208 | Only total sample reported: 22.3(1.9) | 0 | Lifetime suicidal and non-suicidal self-harm: files and interviews  Dual harm: based on responses to above measure | Mental disorder: Structured Clinical Interview guides for Axis I and II disorders and file information  Symptoms of autism spectrum disorders and other neurodevelopmental disorders: Asperger syndrome/high functioning autism diagnostic interview and structured DSM-IV interview protocol | The dual harm group had significantly more childhood attention deficit symptoms, adult attention deficit symptoms, and adult hyperactivity disorder symptoms than the aggression alone group  There was no significant difference in childhood hyperactivity disorder symptoms |
| Lidberg, Belfrage, Bertilsson, Mattila Evenden, & Åsberg (2000) | Sweden | Cross-sectional | Male homicide offenders:  1: Those who had engaged in dual harm – 12  2: Those who had perpetrated homicide alone – 23 | Not reported | 0 | Suicide attempts (timescale not specified): forensic psychiatric reports  Dual harm: cross-tabulation of responses to the above measure | Personality: The Eysenck Personality Inventory, Eysenck Personality Questionnaire, Marke-Nyman Temperament Scale, Gough Delinquency Scale | There were no significant differences in personality |
| Richmond-Rakerd, Caspi, Arseneault, Baldwin, Danese, Houts, Matthews, Wertz, & Moffitt, (2019) | UK | Longitudinal twin | Twins of the E-Risk Longitudinal Twin Study:  1: Those who had engaged in dual harm - 97  2: Those who had engaged in self-harm alone - 177  3: Those who had engaged in aggression alone – not reported  4: Those who had engaged in no harmful behaviours – 1,475 | Not applicable | Dual harm: 43  Self-harm alone: 76  Gender for no harmful behaviours and aggression alone group not reported | Self-harm, irrespective of suicidal intent:  life history calendar used to aid recall of self-reported self-harm behaviour since age 12  Aggression: official police records during ages 10-22 and self-report questionnaire assessing past-year offending behaviour. Violent offenses  included the use of force or threat of force upon a victim  Dual harm: cross-tabulation of responses to the above measures | Mental health difficulties: DSM-IV based symptoms/ diagnosis of post-traumatic stress disorder, depression, psychosis, and substance dependence. No information on how this data was collected.  Personality: Informant reported Big Five Inventory    Self-regulation:  Caregiver responded to questions from the Shedler-Westen Assessment Procedure 200-item Q-Sort for Adolescents. Teachers responded to unvalidated questionnaire  Self-control: Based on 9 measures, including observational ratings, parent and teacher reports, self-reports and interview judgements | The dual harm group did not significantly differ from the self-harm alone group in childhood depression, childhood anxiety, or risk of developing post-traumatic stress disorder or depression. However, they were distinguished by a significantly higher prevalence of psychotic symptoms and  more likely to meet criteria for alcohol and cannabis dependence  Compared to the aggression alone group, the dual harm group exhibited significantly higher rates of childhood depression and all adolescent mental health difficulties  Low childhood self-control significantly predicted increased odds of engaging in dual harm compared to those who engaged in self-harm alone  Children who were rated by caregivers and teachers as having more self-regulation difficulties were significantly more likely to be in the dual harm group than the self-harm alone group  The dual harm group were distinguished by significantly lower openness, lower conscientiousness and lower agreeableness. They were also significantly higher on extraversion  Compared to those in the no harmful behaviours group, those in the dual harm group were significantly higher on neuroticism and lower in conscientiousness and agreeableness  Compared with the aggression alone group, the dual harm group were significantly lower in conscientiousness and higher in neuroticism |
| Stålenheim (2001) | Sweden | Cross-sectional | Forensic psychiatric male patients:  1: Those who had engaged in dual harm – 11  2. Those who had engaged in self-harm alone – 12  3: Those who had engaged in aggression alone – 15  4: Those who had engaged in no harmful behaviours - 20 | Only total sample reported:  34(11) | 0 | Suicidal behaviour: based on SCID interviews and filed information from the forensic psychiatric assessments. Timeline not specified.    Repeated violent criminality: identified from participants’ registered violent criminality. Those sentenced three or more times were identified as showing repeated violent criminality. Timeline not specified  Dual harm: cross-tabulation of responses to the above measures | Personality: Karolinska Scales of Personality | No significant differences in the psychopathy and aggression-related scales  Compared to the aggression alone group, the dual harm group scored significantly higher on psychopathy, aggression and hostility factors |
| Steeg, Webb, Mok, Pedersen, Antonsen, Kapur, & Carr (2019) | Denmark | Nested case-control | Cohort of individuals born to Danish native parents, alive and residing in Denmark on their 15th birthday:  1: Those who had engaged in dual harm - 145  2: Those who had engaged in self-harm alone - 287  3: Those who had engaged in aggression alone - 228 | Age range: 15-35. Median at time of death: 21.1 | Only total sample reported: 28.7 | Hospital treated self-harm episodes since age of 10, irrespective of suicidal intent: [identified from National Patient Register and the Psychiatric Central Research Register](https://www.sciencedirect.com/science/article/pii/S2468266719300428#bib22)  Violent crime since age of 15, including threats to safety and intimidation, as well as physical assaults: identified from National Crime Register  Dual harm: cross-tabulation of responses to the above measures | Substance misuse, psychiatric disorder: data from Psychiatric Central Research Register | Among those who died from any external cause, the prevalence of substance use disorder was higher among the dual harm group compared to those in the self-harm alone and aggression alone groups  There were no significant differences between the groups in regard to other psychiatric disorders |
| Steinhoff, Bechtiger, Ribeaud, Eisner, & Shanahan (2022) | Switzerland | Longitudinal | Sample of first-graders attending public school from the Zurich Project on Social Development from Childhood to Adulthood:  1. Those who had engaged in dual harm – 107  2. Those who had engaged in self-harm alone – 240  3. Those who had engaged in aggression alone - 197 | Not applicable | Dual harm: 7.2  Self-harm alone: 16.2  Aggression alone: 13.3 | Self-harm, irrespective of suicidal intent: self-reported at ages 13, 15 and 17 using one item  Aggression: response to an item from a broader delinquency scale, reported at 13, 15, and 17  Dual harm: cross-tabulation of responses to the above measures | Anxiety/depression at age 20: Social Behaviour Questionnaire  Self-control at age 20: Self-Control Scale  Psychopathy at age 20: The Short Dark Triad  Substance use at age 20: 14 item questionnaire asking about past year substance use (Quednow et al., 2021; Shanahan et al., 2021) | Adjusted associations between behavioural groups at age 13-17 and psychological factors at age 20, controlling for sex, parental educational and migration background, and child’s education level at age 13: compared to no harm, self-harm alone and aggression alone groups, dual harm group reported more anxiety/depression and psychopathy symptoms. Dual harm group also scored significantly higher on substance use and lack of self-control compared to no harm group. |
| Swogger, Walsh, Homaifar, Caine, & Conner (2012) | USA | Longitudinal | Civil admission psychiatric patients:  1: Those who had engaged in dual harm - 94  2: Those who had engaged in self-harm alone - 149  3: Those who had engaged in aggression alone - 144  4: Those who had engaged in no harmful behaviours - 464 | Dual harm: 28.6(5.2)  Self-harm alone: 29.6(6.2)  Aggression alone: 29.7(6.5)  No harmful behaviours: 30.3(6.3) | Dual-harm: 44  Self-harm alone: 61  Aggression alone: 31  No harmful behaviours: 39 | Self-harm, irrespective of suicidal intent: interview asking about self-harm behaviour during 10 weeks since the previous interview  Aggression defined as battery resulting in physical injury, sexual assault, and threats with weapons in hand: assessed in follow-up interview and interviews with collateral informants. Not clear whether this was also assessed in the preceding 10 weeks  Dual harm: cross-tabulation of responses to the above measures | Substance use disorder diagnosis: DSM-III-R checklist  Psychopathy: PCL:SV  Anger: Novaco Anger Scale | Unadjusted analysis: compared to the no harmful behaviours group, substance use disorder, anger and each psychopathy facet were significantly positively associated with dual harm  Analysis adjusted for covariates: compared to no harmful behaviours group, anger and the antisocial facet of psychopathy predicted dual harm |
| Tardiff (1981) | USA | Cross-sectional | Inpatients at psychiatric hospital:  1: Those who had engaged in dual harm - 42  2: Those who had engaged in self-harm alone - 52 | Not reported | Only reported for all patients: 60% | Suicidal behaviour and physical aggression towards others in past 3 months: standardised measure reported by staff  Dual harm: cross-tabulation of responses to the above measures | Psychopathology: adapted NOSIE scale | No significant differences in psychopathology |
| Watkins, Sippel, Pietrzak, Hoff, & Harpaz-Rotem (2017) | USA | Cross-sectional | Veterans in residential treatment programme for post-traumatic stress disorder:  1: Those who had engaged in dual harm - 202  2: Those who had engaged in no harmful behaviours - 856  3: Those who had engaged in aggression alone – 1,471  4: Those who had engaged in self-harm alone - 41 | Dual harm: 39.97(13.05)  Self-harm alone: 45.83(13.62)  Aggression alone: 44.06(14.07)  No harmful behaviour: 50.30(13.86) | Dual harm: 6  Self-harm alone: 5  Aggression alone: 5  No harmful behaviour: 11 | Suicide attempt in past 4 months: one self-report question    Aggression in past 4 months: self-report measure based on items in National Vietnam Readjustment Study. Items included destroyed property, threatened someone with physical violence, had physical fight, threatened someone with weapon  Dual harm: cross-tabulation of responses to the above measures | Post-traumatic stress disorder symptoms: PTSD Checklist-Civilian for DSM-IV | More severe re-experiencing symptoms were related to a significantly higher probability of engaging in dual harm compared to no harmful behaviours  Greater dysphoric arousal symptoms was related to a significantly higher probability of engaging in dual harm compared to no harmful behaviours  Compared to dual harm, greater dysphoric arousal was significantly associated with a lower probability of engaging in self-harm alone  Compared to dual harm, greater re-experiencing symptoms were significantly associated with a lower probability of engaging in aggression alone |

Appendix C. Adapted risk of bias assessment

Unbiased selection of the cohort: As described in tool.

Selection minimizes baseline differences in demographic factors: As described in tool.

Sample size calculated: This criterion was adapted so that studies that did not provide a justification for their sample size but had a sample size of 1000 or over also met the criteria

Adequate description of the cohort: As described in tool.

*Validating method for ascertaining clinical status or participant group; validated methods for assessing outcome or criterion variable :* These two categories were combined to make one category - “*Validated method for ascertaining harmful behaviour group”* as the outcome (e.g., dual harm) and participant group (e.g., those who engage in dual harm) were the same. The criteria for the original categories were combined for the new category. We also clarified that official records (e.g., official national databases, hospital treated self-harm episodes) were valid methods for ascertaining harmful behaviours. If only one harmful behaviour variable was assessed using valid methods, the study would be rated as partially meeting the criteria.

*Validated methods for assessing predictor or risk variables:* This category was adapted so that if only one harmful behaviour variable was assessed using valid methods, the study would be rated as partially meeting the criteria.

*Outcome assessments blind to diagnostic/clinical/participant status:* As described in tool.

*Adequate follow-up period (longitudinal studies only):* As described in tool.

*Missing data is minimal:* As described in tool.

*Analysis controls for confounding:* As described in tool.

*Analytic methods appropriate:* As described in tool.

Appendix D. Excluded articles

| **Reference** | **Primary reason for excluding** |
| --- | --- |
| Centers for Disease Control and Prevention. (1991). Homicide followed by suicide--Kentucky, 1985-1990. *MMWR: Morbidity and mortality weekly report*, *40*(38), 652-3. | Case study |
| Abolarin, J., McLafferty, L., Carmichael, H., & Velopulos, C. G. (2019). Family can hurt you the most: Examining perpetrators in multiple casualty events. *Journal of surgical research*, *242*, 172-176. | No eligible psychological factors reported in relation to the dual-harm group |
| Adhia, A., DeCou, C. R., Huppert, T., & Ayyagari, R. (2020). Murder–Suicides Perpetrated by Adolescents: Findings from the National Violent Death Reporting System. *Suicide and Life‐Threatening Behavior*, *50*(2), 534-544. | Only descriptive statistics reported |
| Adhia, A., Kernic, M. A., Hemenway, D., Vavilala, M. S., & Rivara, F. P. (2019). Intimate partner homicide of adolescents. *JAMA pediatrics*, *173*(6), 571-577. | Dual-harm behaviour was not reported as an outcome |
| Adinkrah, M. (2003). Homicide—Suicides in Fiji: Offense Patterns, Situational Factors, and Sociocultural Contexts. *Suicide and Life-Threatening Behavior*, *33*(1), 65-73. | No eligible psychological factors reported in relation to the dual-harm group |
| Adinkrah, M. (2014). Intimate partner femicide–suicides in Ghana: Victims, offenders, and incident characteristics. *Violence Against Women*, *20*(9), 1078-1096. | No eligible psychological factors reported in relation to the dual-harm group |
| Ağargün, M. Y., Beşiroğlu, L., Güleç, M., Aydın, A., & Selvi, Y. (2016). Sleep-related violence, self-mutilation, and dissociative experiences. | Dual-harm behaviour was not reported as an outcome |
| Allen, D., Bethell, K., & Allen-Carroll, M. (2017). Anger and social fragmentation: The evil violence tunnel. *Journal of Psychotherapy Integration*, *27*(1), 79. | Dual-harm behaviour was not reported as an outcome |
| Apter, A., Plutchik, R., Sevy, S., Korn, M. L., Brown, S., & Van Praag, H. (1989). Defense mechanisms in risk of suicide and risk of violence. *The American journal of psychiatry*. | Dual-harm behaviour was not reported as an outcome |
| Apter, A., Plutchik, R., & Van Praag, H. M. (1993). Anxiety, impulsivity and depressed mood in relation to suicidal and violent behavior. *Acta Psychiatrica Scandinavica*, *87*(1), 1-5. | Dual-harm behaviour was not reported as an outcome |
| Ash, D., Haynes, J., Braben, P., & Galletly, C. (2003). Violence, self-harm, victimisation and homelessness in patients admitted to an acute inpatient unit in South Australia. *International journal of social psychiatry*, *49*(2), 112-118. | Dual-harm behaviour was not reported as an outcome |
| Asnis, G. M., Kaplan, M. L., van Praag, H. M., & Sanderson, W. C. (1994). Homicidal behaviors among psychiatric outpatients. *Psychiatric Services*, *45*(2), 127-132. | Dual-harm behaviour was not reported as an outcome |
| Balica, E., & Stöckl, H. (2016). Homicide–suicides in Romania and the role of migration. *European Journal of Criminology*, *13*(4), 517-534. | No eligible psychological factors reported in relation to the dual-harm group |
| Banks, L., Crandall, C., Sklar, D., & Bauer, M. (2008). A comparison of intimate partner homicide to intimate partner homicide–suicide: one hundred and twenty-four New Mexico cases. *Violence Against Women*, *14*(9), 1065-1078. | No eligible psychological factors reported in relation to the dual-harm group |
| Barber, J. W., Hundley, P., Kellogg, E., Glick, J. L., Godleski, L., Kerler, R., & Vieweg, W. V. R. (1988). Clinical and demographic characteristics of 15 patients with repetitively assaultive behavior. *Psychiatric Quarterly*, *59*(3), 213-224. | Dual-harm behaviour was not reported as an outcome |
| Biro, M., Selakovic-Buršic, S., & Kapamadzija, B. (1991). The role of depressive disorder in the suicidal behavior of alcoholics. *Crisis: The Journal of Crisis Intervention and Suicide Prevention*. | Full text could not be found and could not find contact information for authors |
| Bossarte, R. M., Simon, T. R., & Barker, L. (2006). Characteristics of homicide followed by suicide incidents in multiple states, 2003–04. *Injury prevention*, *12*(suppl 2), ii33-ii38. | Dual-harm behaviour was not reported as an outcome |
| Botsis, A. J., Plutchik, R., Kotler, M., & van Praag, H. M. (1995). Parental loss and family violence as correlates of suicide and violence risk. *Suicide and Life‐Threatening Behavior*, *25*(2), 253-260. | Dual-harm behaviour was not reported as an outcome |
| Bourget, D., Gagne, P., & Moamai, J. (2000). Spousal homicide and suicide in Quebec. *Journal of the American Academy of Psychiatry and the Law Online*, *28*(2), 179-182. | No eligible psychological factors reported in relation to the dual-harm group |
| Bourget, D., & Gagné, P. (2005). Paternal filicide in Quebec. *Journal of the American Academy of Psychiatry and the Law Online*, *33*(3), 354-360. | Only descriptive statistics reported |
| Bourget, D., Gagné, P., & Labelle, M. E. (2007). Parricide: a comparative study of matricide versus patricide. *Journal of the American Academy of Psychiatry and the Law Online*, *35*(3), 306-312. | Only descriptive statistics reported |
| Bourget, D., Gagné, P., & Whitehurst, L. (2010). Domestic homicide and homicide-suicide: The older offender. *Journal of the American Academy of Psychiatry and the Law Online*, *38*(3), 305-311. | Only descriptive statistics reported |
| Bourget, D., & Gagné, P. (2012). Women who kill their mates. *Behavioral sciences & the law*, *30*(5), 598-614. | Only descriptive statistics reported |
| Bouvy, P. F., & Liem, M. (2012). Antidepressants and lethal violence in the Netherlands 1994–2008. *Psychopharmacology*, *222*(3), 499-506. | Dual-harm behaviour was not reported as an outcome |
| Branchey, L., Branchey, M., Shaw, S., & Lieber, C. S. (1984). Depression, suicide, and aggression in alcoholics and their relationship to plasma amino acids. *Psychiatry research*, *12*(3), 219-226. | Dual-harm behaviour was not reported as an outcome |
| Brylewski, J., & Wiggs, L. (1999). Sleep problems and daytime challenging behaviour in a community‐based sample of adults with intellectual disability. *Journal of Intellectual Disability Research*, *43*(6), 504-512. | Sample under study had a developmental condition |
| Buchanan, C. B., Stallworth, J. L., Scott, A. E., Glaze, D. G., Lane, J. B., Skinner, S. A., ... & Kaufmann, W. E. (2019). Behavioral profiles in Rett syndrome: data from the natural history study. *Brain and Development*, *41*(2), 123-134. | Dual-harm behaviour was not reported as an outcome |
| Burgess, A. W., Sekula, L. K., & Carretta, C. M. (2015). Homicide-suicide and duty to warn. *Psychodynamic psychiatry*, *43*(1), 67-90. | No eligible psychological factors reported in relation to the dual-harm group |
| Byard, R. W., Knight, D., James, R. A., & Gilbert, J. (1999). Murder-suicides involving children: a 29-year study. *The American Journal of Forensic Medicine and Pathology*, *20*(4), 323-327. | No eligible psychological factors reported in relation to the dual-harm group |
| Cailhol, L., Moraru, R., Barbe, R., Moncany, A. H., Garcin, S., Lazignac, C., & Damsa, C. (2009). Month of birth, a risk factor for violent behaviour in suicidal patients admitted in emergency?. *Psychiatric quarterly*, *80*(2), 125-130. | Dual-harm behaviour was not reported as an outcome |
| Calegaro, V. C., Zatti, C., Bastos, A. G., & Freitas, L. H. M. (2018). Suicidal patients in a psychiatric emergency unit: clinical characteristics and aggression profile. *Trends in psychiatry and psychotherapy*, *41*, 9-17. | Dual-harm behaviour was not reported as an outcome |
| Ciani, A. S. C., & Fontanesi, L. (2012). Mothers who kill their offspring: testing evolutionary hypothesis in a 110-case Italian sample. *Child abuse & neglect*, *36*(6), 519-527. | Dual-harm behaviour was not reported as an outcome |
| Carballo, J. J., García-Nieto, R., Harkavy-Friedman, J., de Leon-Martinez, V., & Baca-García, E. (2014). Aggressiveness across development and suicidal behavior in depressed patients. *Archives of suicide research*, *18*(1), 39-49. | Dual-harm behaviour was not reported as an outcome |
| Carmichael, H., Jamison, E., Bol, K. A., McIntyre Jr, R., & Velopulos, C. G. (2018). Premeditated versus “passionate”: patterns of homicide related to intimate partner violence. *Journal of surgical research*, *230*, 87-93. | Dual-harm behaviour was not reported as an outcome |
| Carretta, C. M., Burgess, A. W., & Welner, M. (2015). Gaps in crisis mental health: suicide and homicide–suicide. *Archives of psychiatric nursing*, *29*(5), 339-345. | No eligible psychological factors reported in relation to the dual-harm group |
| Casner, J. A., Weinheimer, B., & Gualtieri, C. T. (1996). Naltrexone and self-injurious behavior: a retrospective population study. *Journal of clinical psychopharmacology*, *16*(5), 389-394. | Dual-harm behaviour was not reported as an outcome |
| Castelpietra, G., Egidi, L., Caneva, M., Gambino, S., Feresin, T., Mariotto, A., ... & Marzano, L. (2018). Suicide and suicides attempts in Italian prison epidemiological findings from the “Triveneto” area, 2010–2016. *International journal of law and psychiatry*, *61*, 6-12. | Dual-harm behaviour was not reported as an outcome |
| Choi, N. G., DiNitto, D. M., & Marti, C. N. (2019). Suicide decedents in correctional settings: mental health treatment for suicidal ideation, plans, and/or attempts. *Journal of correctional health care*, *25*(1), 70-83. | Dual-harm behaviour was not reported as an outcome |
| Cohen, D., Llorente, M., & Eisdorfer, C. (1998). Homicide-suicide in older persons. *American Journal of Psychiatry*, *155*(3), 390-396. | Only descriptive statistics reported |
| Cooper, M., & Eaves, D. (1996). Suicide following homicide in the family. *Violence and Victims*, *11*(2), 99-112. | Only descriptive statistics reported |
| Daffern, M., & Howells, K. (2007). The prediction of imminent aggression and self-harm in personality disordered patients of a high security hospital using the HCR-20 clinical scale and the dynamic appraisal of situational aggression. *International Journal of Forensic Mental Health*, *6*(2), 137-143. | Dual-harm behaviour was not reported as an outcome |
| Daffern, M., & Howells, K. (2009). Self-harm and aggression in dangerous and severely personality disordered patients of a high-security hospital. *Psychiatry, Psychology and Law*, *16*(1), 150-154. | No eligible psychological factors reported in relation to the dual-harm group |
| Dharmawardene, V., & Menkes, D. B. (2017). Violence and self-harm in severe mental illness: inpatient study of associations with ethnicity, cannabis and alcohol. *Australasian psychiatry*, *25*(1), 28-31. | Dual-harm behaviour was not reported as an outcome |
| Di Lorenzo, R., Sagona, M., Landi, G., Martire, L., Piemonte, C., & Del Giovane, C. (2016). The revolving door phenomenon in an Italian acute psychiatric ward: a 5-year retrospective analysis of the potential risk factors. *The Journal of nervous and mental disease*, *204*(9), 686-692. | Dual-harm behaviour was not reported as an outcome |
| Ducasse, D., Jaussent, I., Olié, E., Guillaume, S., Lopez-Castroman, J., & Courtet, P. (2016). Personality traits of suicidality are associated with premenstrual syndrome and premenstrual dysphoric disorder in a suicidal women sample. *PloS one*, *11*(2), e0148653. | Dual-harm behaviour was not reported as an outcome |
| Duncan, D., Matson, J. L., Bamburg, J. W., Cherry, K. E., & Buckley, T. (1999). The relationship of self-injurious behavior and aggression to social skills in persons with severe and profound learning disability. *Research in developmental disabilities*, *20*(6), 441-448. | Sample under study had a developmental condition |
| Easteal, P. (1994). Homicide‐suicides between adult sexual intimates: an Australian study. *Suicide and Life‐Threatening Behavior*, *24*(2), 140-151. | No eligible psychological factors reported in relation to the dual-harm group |
| Eckert, E. M., Dominick, K. C., Pedapati, E. V., Wink, L. K., Shaffer, R. C., Andrews, H., ... & Erickson, C. A. (2019). Pharmacologic interventions for irritability, aggression, agitation and self-injurious behavior in fragile X syndrome: an initial cross-sectional analysis. *Journal of autism and developmental disorders*, *49*(11), 4595-4602. | Dual-harm behaviour was not reported as an outcome |
| Emerson, E., & Bromley, J. (1995). The form and function of challenging behaviours. *Journal of Intellectual Disability Research*, *39*(5), 388-398. | No eligible psychological factors reported in relation to the dual-harm group |
| Emerson, E., Kiernan, C., Alborz, A., Reeves, D., Mason, H., Swarbrick, R., ... & Hatton, C. (2001). The prevalence of challenging behaviors: A total population study. *Research in developmental disabilities*, *22*(1), 77-93. | Dual-harm behaviour was not reported as an outcome |
| Engström, G., Alsén, M., Gustavsson, P., Schalling, D., & Träskman-Bendz, L. (1996). Classification of suicide attempters by cluster analysis: A study of the temperamental heterogeneity in suicidal patients. *Personality and individual differences*, *21*(5), 687-695. | Dual-harm behaviour was not reported as an outcome |
| Fanning, J. R., Meyerhoff, J. J., Lee, R., & Coccaro, E. F. (2014). History of childhood maltreatment in intermittent explosive disorder and suicidal behavior. *Journal of psychiatric research*, *56*, 10-17. | Dual-harm behaviour was not reported as an outcome |
| Flynn, S., Gask, L., Appleby, L., & Shaw, J. (2016). Homicide–suicide and the role of mental disorder: a national consecutive case series. *Social psychiatry and psychiatric epidemiology*, *51*(6), 877-884. | Only descriptive statistics reported |
| Frei, A., & Ilic, A. (2020). Is familicide a distinct subtype of mass murder? Evidence from a Swiss national cohort. *Criminal behaviour and mental health*, *30*(1), 28-37. | Only descriptive statistics reported |
| Friedman, S. H., Hrouda, D. R., Holden, C. E., Noffsinger, S. G., & Resnick, P. J. (2005). Filicide-suicide: common factors in parents who kill their children and themselves. *Journal of the American Academy of Psychiatry and the Law Online*, *33*(4), 496-504. | No eligible psychological factors reported in relation to the dual-harm group |
| Gazdag, G., Belán, E., Szabó, F. A., Ungvari, G. S., Czobor, P., & Baran, B. (2015). Predictors of suicide attempts after violent offences in schizophrenia spectrum disorders. *Psychiatry research*, *230*(2), 728-731. | No eligible psychological factors reported in relation to the dual-harm group |
| Gelegen, V., & Tamam, L. (2018). Prevalence and clinical correlates of intermittent explosive disorder in Turkish psychiatric outpatients. *Comprehensive psychiatry*, *83*, 64-70. | Dual-harm behaviour was not reported as an outcome |
| Geller, A. M., & Atkins, A. L. (1978). Cognitive and personality factors in suicidal behavior. *Journal of consulting and clinical psychology*, *46*(5), 860. | Dual-harm behaviour was not reported as an outcome |
| Gerard, F. J., Whitfield, K. C., Porter, L. E., & Browne, K. D. (2016). Offender and offence characteristics of school shooting incidents. *Journal of Investigative Psychology and Offender Profiling*, *13*(1), 22-38. | Dual-harm behaviour was not reported as an outcome |
| Gibbons, P., Gannon, M., & Wrigley, M. (1997). A study of aggression among referrals to a community‐based psychiatry of old age service. *International journal of geriatric psychiatry*, *12*(3), 384-388. | Dual-harm behaviour was not reported as an outcome |
| Gibson, K. R. (2011). *The roles of operational sex ratio and young-old ratio in producing suicide attackers*. The University of Utah. | No eligible psychological factors reported in relation to the dual-harm group |
| Gos, T., Krell, D., Bielau, H., Brisch, R., Trübner, K., Steiner, J., ... & Bogerts, B. (2008). Tyrosine hydroxylase immunoreactivity in the locus coeruleus is elevated in violent suicidal depressive patients. *European archives of psychiatry and clinical neuroscience*, *258*(8), 513-520. | Dual-harm behaviour was not reported as an outcome |
| Greenwald, D. J., Reznikoff, M., & Plutchik, R. (1994). Suicide risk and violence risk in alcoholics: Predictors of aggressive risk. *Journal of nervous and mental disease*. | Dual-harm behaviour was not reported as an outcome |
| Greenwald, D. J. (1991). *Suicide and violence risk: Predictors of aggressive expression* (Doctoral dissertation, Fordham University). | Dual-harm behaviour was not reported as an outcome |
| Gregory, M. J., & Milroy, C. M. (2010). Homicide and suicide in Yorkshire and the Humber: 1975–1992 and 1993–2007. *The American journal of forensic medicine and pathology*, *31*(1), 58-63. | Only descriptive statistics reported |
| Haapasalo, J., & Petäjä, S. (1999). Mothers who killed or attempted to kill their child: life circumstances, childhood abuse, and types of killing. *Violence and victims*, *14*(3), 219-239. | Only descriptive statistics reported |
| Hannah, S. G., Turf, E. E., & Fierro, M. F. (1998). Murder-suicide in central Virginia: a descriptive epidemiologic study and empiric validation of the Hanzlick-Koponen typology. *The American journal of forensic medicine and pathology*, *19*(3), 275-283. | Full text could not be found and could not find contact information for authors |
| Harford, T. C., Yi, H. Y., & Grant, B. F. (2013). Other-and self-directed forms of violence and their relationships to DSM-IV substance use and other psychiatric disorders in a national survey of adults. *Comprehensive psychiatry*, *54*(7), 731-739. | Dual-harm behaviour was not reported as an outcome |
| Hart, S. R., Van Eck, K., Ballard, E. D., Musci, R. J., Newcomer, A., & Wilcox, H. C. (2017). Subtypes of suicide attempters based on longitudinal childhood profiles of co-occurring depressive, anxious and aggressive behavior symptoms. *Psychiatry research*, *257*, 150-155. | Dual-harm behaviour was not reported as an outcome |
| Hedlund, J., Masterman, T., & Sturup, J. (2016). Intra-and extra-familial child homicide in Sweden 1992–2012: A population-based study. *Journal of forensic and legal medicine*, *39*, 91-99. | Only descriptive statistics reported |
| Heller, M. S., Ehrlich, S. M., & Lester, D. (1985). Suicidal history of defendants and offenders. *The Journal of general psychology*, *112*(2), 221-223. | Dual-harm behaviour was not reported as an outcome |
| Hillbrand, M. (1995). Aggression against self and aggression against others in violent psychiatric patients. *Journal of consulting and clinical psychology*, *63*(4), 668. | Dual-harm behaviour was not reported as an outcome |
| Hillbrand, M., Krystal, J. H., Sharpe, K. S., & Foster, H. G. (1994). Clinical predictors of self-mutilation in hospitalized forensic patients. *Journal of Nervous and Mental Disease*. | Dual-harm behaviour was not reported as an outcome |
| Hiss, J., & Kahana, T. (1998). Suicide bombers in Israel. *The American journal of forensic medicine and pathology*, *19*(1), 63-66. | Dual-harm behaviour was not reported as an outcome |
| Huguet, N., & Lewis-Laietmark, C. (2015). Rates of homicide-followed-by-suicide among White, African American, and Hispanic men. *Public health*, *129*(3), 280-282. | No eligible psychological factors reported in relation to the dual-harm group |
| Janowsky, D. S., Barnhill, L. J., & Davis, J. M. (2003). Olanzapine for self-injurious, aggressive, and disruptive behaviors in intellectually disabled adults: a retrospective, open-label, naturalistic trial. *Journal of Clinical Psychiatry*, *64*(10), 1258-1265. | Sample under study had a developmental condition |
| Janowsky, D. S., Shetty, M., Barnhill, J., Elamir, B., & Davis, J. M. (2005). Serotonergic antidepressant effects on aggressive, self-injurious and destructive/disruptive behaviours in intellectually disabled adults: a retrospective, open-label, naturalistic trial. *International Journal of Neuropsychopharmacology*, *8*(1), 37-48. | Sample under study had a developmental condition |
| John, C. J. (2000). Family murder suicides in Kerala: A column of Befrienders International. | No eligible psychological factors reported in relation to the dual-harm group |
| Johnson, C., Smith, J., Crowe, C., & Donovan, M. (1993). Suicide among forensic psychiatric patients. *Medicine, Science and the Law*, *33*(2), 137-143. | Case study |
| Johnson, K. L., Desmarais, S. L., Tueller, S. J., Grimm, K. J., Swartz, M. S., & Van Dorn, R. A. (2016). A longitudinal analysis of the overlap between violence and victimization among adults with mental illnesses. *Psychiatry research*, *246*, 203-210. | Dual-harm behaviour was not reported as an outcome |
| Jokinen, J., Forslund, K., Nordström, A. L., Lindqvist, P., & Nordström, P. (2009). Suicide risk after homicide in Sweden. *Archives of suicide research*, *13*(3), 297-301. | No eligible psychological factors reported in relation to the dual-harm group |
| Kalish, R., & Kimmel, M. (2010). Suicide by mass murder: Masculinity, aggrieved entitlement, and rampage school shootings. *Health Sociology Review*, *19*(4), 451-464. | Case study |
| Karch, D., & Nunn, K. C. (2011). Characteristics of elderly and other vulnerable adult victims of homicide by a caregiver: National Violent Death Reporting System—17 US States, 2003-2007. *Journal of Interpersonal Violence*, *26*(1), 137-157. | Dual-harm behaviour was not reported as an outcome |
| Kerry, G. P. (2001). *Understanding and predicting intimate femicide; an analysis of men who kill their intimate female partners* (Doctoral dissertation, Carleton University). | Dual-harm behaviour was not reported as an outcome |
| Khalsa, H. M. K., Salvatore, P., Hennen, J., Baethge, C., Tohen, M., & Baldessarini, R. J. (2008). Suicidal events and accidents in 216 first-episode bipolar I disorder patients: predictive factors. *Journal of affective disorders*, *106*(1-2), 179-184. | No eligible psychological factors reported in relation to the dual-harm group |
| Kingston, J., Clarke, S., Ritchie, T., & Remington, B. (2011). Developing and validating the “composite measure of problem behaviors”. *Journal of clinical psychology*, *67*(7), 736-751. | Dual-harm behaviour was not reported as an outcome |
| Kleiman, E. M., Ammerman, B. A., Kulper, D. A., Uyeji, L. L., Jenkins, A. L., & McCloskey, M. S. (2015). Forms of non-suicidal self-injury as a function of trait aggression. *Comprehensive psychiatry*, *59*, 21-27. | Dual-harm behaviour was not reported as an outcome |
| Klinoff, V. A., Van Hasselt, V. B., & Black, R. A. (2015). Homicide-suicide in police families: an analysis of cases from 2007-2014. *Journal of forensic practice*. | No eligible psychological factors reported in relation to the dual-harm group |
| Knoll, J. L., & Hatters‐Friedman, S. (2015). The homicide–suicide phenomenon: findings of psychological autopsies. *Journal of forensic sciences*, *60*(5), 1253-1257. | Only descriptive statistics reported |
| Korn, M. L., Kotler, M., Molcho, A., Botsis, A. J., Grosz, D., Chen, C., ... & Van Praag, H. M. (1992). Suicide and violence associated with panic attacks. *Biological psychiatry*, *31*(6), 607-612. | Case study |
| Korn, M. L., Plutchik, R., & Van Praag, H. M. (1997). Panic-associated suicidal and aggressive ideation and behavior. *Journal of Psychiatric Research*, *31*(4), 481-487. | Dual-harm behaviour was not reported as an outcome |
| Kottler, C., Smith, J. G., & Bartlett, A. (2018). Patterns of violence and self-harm in women prisoners: characteristics, co-incidence and clinical significance. *The Journal of Forensic Psychiatry & Psychology*, *29*(4), 617-634. | No eligible psychological factors reported in relation to the dual-harm group |
| Koziol-McLain, J., Webster, D., McFarlane, J., Block, C. R., Ulrich, Y., Glass, N., & Campbell, J. C. (2006). Risk factors for femicide-suicide in abusive relationships: Results from a multisite case control study. *Violence and victims*, *21*(1), 3-21. | Comparison group not eligible |
| Krabbendam, A. A., Jansen, L. M., Van De Ven, P. M., Van der Molen, E., Doreleijers, T. A., & Vermeiren, R. R. (2014). Persistence of aggression into adulthood in detained adolescent females. *Comprehensive psychiatry*, *55*(7), 1572-1579. | Dual-harm behaviour was not reported as an outcome |
| Krakowski, M., & Czobor, P. (2004). Suicide and violence in patients with major psychiatric disorders. *Journal of Psychiatric Practice®*, *10*(4), 233-238. | Dual-harm behaviour was not reported as an outcome |
| Krakowski, M. I., Convit, A., & Volavka, J. (1988). Patterns of inpatient assaultiveness: Effect of neurological impairment and deviant family environment on response to treatment. *Neuropsychiatry, Neuropsychology, & Behavioral Neurology*. | Dual-harm behaviour was not reported as an outcome |
| Krischer, M. K., Stone, M. H., Sevecke, K., & Steinmeyer, E. M. (2007). Motives for maternal filicide: Results from a study with female forensic patients. *International journal of law and psychiatry*, *30*(3), 191-200. | Dual-harm behaviour was not reported as an outcome |
| Krulewitch, C. J. (2009). Epidemiology of intimate partner homicide-suicide events among women of childbearing age in Maryland, 1994–2003. *The American journal of forensic medicine and pathology*, *30*(4), 362-365. | No eligible psychological factors reported in relation to the dual-harm group |
| Lambrechts, G., Kuppens, S., & Maes, B. (2009). Staff variables associated with the challenging behaviour of clients with severe or profound intellectual disabilities. *Journal of Intellectual Disability Research*, *53*(7), 620-632. | Sample under study had a developmental condition |
| Lanier, C. (2010). Structure, culture, and lethality: An integrated model approach to American Indian suicide and homicide. *Homicide Studies*, *14*(1), 72-89. | Dual-harm behaviour was not reported as an outcome |
| Lankford, A. (2015). Mass murderers in the United States: Predictors of offender deaths. *The Journal of Forensic Psychiatry & Psychology*, *26*(5), 586-600. | Dual-harm behaviour was not reported as an outcome |
| Lecomte, D., & Fornes, P. (1998). Homicide followed by suicide: Paris and its suburbs, 1991–1996. *Journal of Forensic Science*, *43*(4), 760-764. | No psychological factors reported in relation to the dual-harm group |
| Leenaars, A. A. (1989). Suicide across the adult life-span: an archival study. *Crisis: The Journal of Crisis Intervention and Suicide Prevention*. | Dual-harm behaviour was not reported as an outcome |
| Lester, D. (1967). Suicide as an aggressive act. *The Journal of psychology*, *66*(1), 47-50. | Dual-harm behaviour was not reported as an outcome |
| Lester, D. (1988). Religion and personal violence (homicide and suicide) in the USA. *Psychological reports*, *62*(2), 618-618. | Dual-harm behaviour was not reported as an outcome |
| Lester, D. (2014). Murder-suicide in workplace violence. *Psychological reports*, *115*(1), 28-31. | No eligible psychological factors reported in relation to the dual-harm group |
| Lewis, M. H., Silva, J. R., & G-Silva, S. (1995). Cyclicity of aggression and self-injurious behavior in individuals with mental retardation. *American journal on mental retardation*. | Sample under study had a developmental condition |
| Liem, M., Barber, C., Markwalder, N., Killias, M., & Nieuwbeerta, P. (2011). Homicide–suicide and other violent deaths: An international comparison. *Forensic Science International*, *207*(1-3), 70-76. | No eligible psychological factors reported in relation to the dual-harm group |
| Liem, M., & Nieuwbeerta, P. (2010). Homicide followed by suicide: a comparison with homicide and suicide. *Suicide and Life-Threatening Behavior*, *40*(2), 133-145. | No eligible psychological factors reported in relation to the dual-harm group |
| Liettu, A., Mikkola, L., Säävälä, H., Räsänen, P., Joukamaa, M., & Hakko, H. (2010). Mortality rates of males who commit parricide or other violent offense against a parent. *Journal of the American Academy of Psychiatry and the Law Online*, *38*(2), 212-220. | Dual-harm behaviour was not reported as an outcome |
| Logan, J. E., Walsh, S., Patel, N., & Hall, J. E. (2013). Homicide-followed-by-suicide incidents involving child victims. *American journal of health behavior*, *37*(4), 531-542. | Only descriptive statistics reported |
| Logan, J., Hall, J., & Karch, D. (2011). Suicide categories by patterns of known risk factors: a latent class analysis. *Archives of general psychiatry*, *68*(9), 935-941. | Dual-harm behaviour was not reported as an outcome |
| Long, C. G., Fulton, B., Dolley, O., & Hollin, C. R. (2011). Dealing with feelings: The effectiveness of cognitive behavioural group treatment for women in secure settings. *Behavioural and cognitive psychotherapy*, *39*(2), 243-247. | Dual-harm behaviour was not reported as an outcome |
| Lund, L. E., & Smorodinsky, S. (2001). Violent death among intimate partners: A comparison of homicide and homicide followed by suicide in California. *Suicide and Life-Threatening Behavior*, *31*(4), 451-459. | Dual-harm behaviour was not reported as an outcome |
| Lundqvist, L. O. (2011). Psychometric properties and factor structure of the Behavior Problems Inventory (BPI-01) in a Swedish community population of adults with intellectual disability. *Research in developmental disabilities*, *32*(6), 2295-2303. | Dual-harm behaviour was not reported as an outcome |
| Malphurs, J. E., & Cohen, D. (2005). A statewide case–control study of spousal homicide–suicide in older persons. *The American Journal of Geriatric Psychiatry*, *13*(3), 211-217. | Only descriptive statistics reported |
| Manning, J. (2015). The social structure of homicide-suicide. *Homicide Studies*, *19*(4), 350-369. | No eligible psychological factors reported in relation to the dual-harm group |
| Maremmani, A. G., Cerniglia, L., Cimino, S., Bacciardi, S., Rovai, L., Pallucchini, A., ... & Maremmani, I. (2017). Further evidence of a specific psychopathology of addiction. Differentiation from other psychiatric psychopathological dimensions (such as obesity). *International journal of environmental research and public health*, *14*(8), 943. | Dual-harm behaviour was not reported as an outcome |
| Marttunen, M. J., Aro, H. M., Henriksson, M. M., & Lönnqvist, J. K. (1994). Antisocial behaviour in adolescent suicide. *Acta Psychiatrica Scandinavica*, *89*(3), 167-173. | Sample not adults |
| Mathews, S., Abrahams, N., Jewkes, R., Martin, L. J., Lombard, C., & Vetten, L. (2008). Intimate femicide-suicide in South Africa: a cross-sectional study. *Bulletin of the World Health Organization*, *86*, 542-558. | No eligible psychological factors reported in relation to the dual-harm group |
| Matson, J. L., Cooper, C., Malone, C. J., & Moskow, S. L. (2008). The relationship of self-injurious behavior and other maladaptive behaviors among individuals with severe and profound intellectual disability. *Research in Developmental Disabilities*, *29*(2), 141-148. | Sample under study had a developmental condition |
| McCloskey, M. S., Ben-Zeev, D., Lee, R., & Coccaro, E. F. (2008). Prevalence of suicidal and self-injurious behavior among subjects with intermittent explosive disorder. *Psychiatry research*, *158*(2), 248-250. | Dual-harm behaviour was not reported as an outcome |
| McPhedran, S., Eriksson, L., Mazerolle, P., De Leo, D., Johnson, H., & Wortley, R. (2018). Characteristics of homicide-suicide in Australia: a comparison with homicide-only and suicide-only cases. *Journal of interpersonal violence*, *33*(11), 1805-1829. | No eligible psychological factors reported in relation to the dual-harm group |
| Mehlum, L. (1992). Prodromal signs and precipitating factors in attempted suicide. *Military medicine*, *157*(11), 574-577. | Dual-harm behaviour was not reported as an outcome |
| Modestin, J., & Emmenegger, P. A. (1986). Completed suicide and criminality: lack of a direct relationship. *Psychological medicine*, *16*(3), 661-669. | Dual-harm behaviour was not reported as an outcome |
| Mok, P. L., Pedersen, C. B., Springate, D., Astrup, A., Kapur, N., Antonsen, S., ... & Webb, R. T. (2016). Parental psychiatric disease and risks of attempted suicide and violent criminal offending in offspring: a population-based cohort study. *Jama Psychiatry*, *73*(10), 1015-1022. | Dual-harm behaviour was not reported as an outcome |
| Monahan, J., Vesselinov, R., Robbins, P. C., & Appelbaum, P. S. (2017). Violence to others, violent self-victimization, and violent victimization by others among persons with a mental illness. *Psychiatric services*, *68*(5), 516-519. | Comparison group not eligible |
| Morton, E., Runyan, C. W., Moracco, K. E., & Butts, J. (1998). Partner homicide-suicide involving female homicide victims: a population-based study in North Carolina, 1988–1992. *Violence and Victims*, *13*(2), 91-106. | No eligible psychological factors reported in relation to the dual-harm group |
| Nijman, H. L., & à Campo, J. M. (2002). Situational determinants of inpatient self-harm. *Suicide and Life-Threatening Behavior*, *32*(2), 167-175. | No eligible psychological factors reported in relation to the dual-harm group |
| Oliffe, J. L., Han, C. S., Drummond, M., Sta. Maria, E., Bottorff, J. L., & Creighton, G. (2015). Men, masculinities, and murder-suicide. *American journal of men's health*, *9*(6), 473-485. | No eligible psychological factors reported in relation to the dual-harm group |
| Parker, G., Roy, K., Wilhelm, K., Austin, M. P., Mitchell, P., & Hadzi-Pavlovic, D. (1998). “Acting out” and “acting in” as behavioral responses to stress: a qualitative and quantitative study. *Journal of personality disorders*, *12*(4), 338-350. | Dual-harm behaviour was not reported as an outcome |
| Patel, V., & de Moore, G. M. (1994). Harakiri: a clinical study of deliberate self-stabbing. *The Journal of clinical psychiatry*. | Dual-harm behaviour was not reported as an outcome |
| Patton, C. L., McNally, M. R., & Fremouw, W. J. (2017). Military versus civilian murder-suicide. *Journal of interpersonal violence*, *32*(17), 2566-2590. | Comparison group not eligible |
| Payne, J., Malla, A., Norman, R., Windell, D., & Brown, N. (2006). Status of first-episode psychosis patients presenting for routine care in a defined catchment area. *The Canadian Journal of Psychiatry*, *51*(1), 42-47. | Dual-harm behaviour was not reported as an outcome |
| Pereira, A. R., Vieira, D. N., & Magalhães, T. (2013). Fatal intimate partner violence against women in Portugal: a forensic medical national study. *Journal of forensic and legal medicine*, *20*(8), 1099-1107. | Dual-harm behaviour was not reported as an outcome |
| Poland, J. M. (2003). Suicide bombers: A global problem. *Humboldt Journal of Social Relations*, 100-135. | No eligible psychological factors reported in relation to the dual-harm group |
| Powell, G., Caan, W., & Crowe, M. (1994). What events precede violent incidents in psychiatric hospitals?. *The British Journal of Psychiatry*, *165*(1), 107-112. | Dual-harm behaviour was not reported as an outcome |
| Pritchard, C., & Bagley, C. (2001). Suicide and murder in child murderers and child sexual abusers. *Journal of Forensic Psychiatry*, *12*(2), 269-286. | Only descriptive statistics reported |
| Pritchard, C., & King, E. (2005). Differential suicide rates in typologies of child sex offenders in a 6-year consecutive cohort of male suicides. *Archives of suicide research*, *9*(1), 35-43. | No eligible psychological factors reported in relation to the dual-harm group |
| Raymond, S., Leger, A. S., & Lachaux, B. (2015). A descriptive and follow-up study of 40 parricidal patients hospitalized in a French secure unit over a 15-year period. *International journal of law and psychiatry*, *41*, 43-49. | Dual-harm behaviour was not reported as an outcome |
| Razali, S., Salleh, R. I. M., Yahya, B., & Ahmad, S. H. (2015). Maternal filicide among women admitted to forensic psychiatric institutions in Malaysia: case series. *East Asian archives of psychiatry*, *25*(2), 79-87. | No eligible psychological factors reported in relation to the dual-harm group |
| Rebok, F., Teti, G. L., Fantini, A. P., Cárdenas-Delgado, C., Rojas, S. M., Derito, M. N., & Daray, F. M. (2015). Types of borderline personality disorder (BPD) in patients admitted for suicide-related behavior. *Psychiatric quarterly*, *86*(1), 49-60. | Dual-harm behaviour was not reported as an outcome |
| Reckdenwald, A., & Simone, S. (2017). Injury patterns for homicide followed by suicide by the relationship between victims and offenders. *Homicide studies*, *21*(2), 111-132. | No eligible psychological factors reported in relation to the dual-harm group |
| Repo, E., Virkkunen, M., Rawlings, R., & Linnoila, M. (1997). Suicidal behavior among Finnish fire setters. *European archives of psychiatry and clinical neuroscience*, *247*(6), 303-307. | Dual-harm behaviour was not reported as an outcome |
| Reynolds, F. M., & Berman, A. L. (1995). An empirical typology of suicide. *Archives of suicide research*, *1*(2), 97-109. | Dual-harm behaviour was not reported as an outcome |
| Roberts, K., Wassenaar, D., Canetto, S. S., & Pillay, A. (2010). Homicide-suicide in Durban, South Africa. *Journal of Interpersonal Violence*, *25*(5), 877-899. | No eligible psychological factors reported in relation to the dual-harm group |
| Rougé-Maillart, C., Jousset, N., Gaudin, A., Bouju, B., & Penneau, M. (2005). Women who kill their children. *The American journal of forensic medicine and pathology*, *26*(4), 320-326. | No eligible psychological factors reported in relation to the dual-harm group |
| Ruedrich, S. L., Swales, T. P., Rossvanes, C., Diana, L., Arkadiev, V., & Lim, K. (2008). Atypical antipsychotic medication improves aggression, but not self‐injurious behaviour, in adults with intellectual disabilities. *Journal of Intellectual Disability Research*, *52*(2), 132-140. | No eligible psychological factors reported in relation to the dual-harm group |
| Sachmann, M., & Harris Johnson, C. M. (2014). The relevance of long‐term antecedents in assessing the risk of familicide‐suicide following separation. *Child abuse review*, *23*(2), 130-141. | Review |
| Sahlin, H., Kuja-Halkola, R., Bjureberg, J., Lichtenstein, P., Molero, Y., Rydell, M., ... & Hellner, C. (2017). Association between deliberate self-harm and violent criminality. *JAMA psychiatry*, *74*(6), 615-621. | Dual-harm behaviour was not reported as an outcome |
| Sahlin, H., Moberg, T., Hirvikoski, T., & Jokinen, J. (2015). Non-suicidal self-injury and interpersonal violence in suicide attempters. *Archives of suicide research*, *19*(4), 500-509. | Dual-harm behaviour was not reported as an outcome |
| Salari, S. (2007). Patterns of intimate partner homicide suicide in later life: Strategies for prevention. *Clinical Interventions in Aging*, *2*(3), 441. | Only descriptive statistics reported |
| Salari, S., & Sillito, C. L. (2016). Intimate partner homicide–suicide: Perpetrator primary intent across young, middle, and elder adult age categories. *Aggression and Violent Behavior*, *26*, 26-34. | No eligible psychological factors reported in relation to the dual-harm group |
| SanSegundo, M. S., Ferrer-Cascales, R., Bellido, J. H., Bravo, M. P., Oltra-Cucarella, J., & Kennedy, H. G. (2018). Prediction of violence, suicide behaviors and suicide ideation in a sample of institutionalized offenders with schizophrenia and other psychosis. *Frontiers in psychology*, *9*, 1385. | Dual-harm behaviour was not reported as an outcome |
| Sani, G., Tondo, L., Koukopoulos, A., Reginaldi, D., Kotzalidis, G. D., Koukopoulos, A. E., ... & Tatarelli, R. (2011). Suicide in a large population of former psychiatric inpatients. *Psychiatry and clinical neurosciences*, *65*(3), 286-295. | Dual-harm behaviour was not reported as an outcome |
| Sansone, R. A., Elliott, K., & Wiederman, M. W. (2016). Self-harm behaviors among female perpetrators of intimate partner violence. *Partner abuse*, *7*(1), 44-54. | Dual-harm behaviour was not reported as an outcome |
| Schretlen, D. J., Ward, J., Meyer, S. M., Yun, J., Puig, J. G., Nyhan, W. L., ... & Harris, J. C. (2005). Behavioral aspects of Lesch–Nyhan disease and its variants. *Developmental Medicine & Child Neurology*, *47*(10), 673-677. | Dual-harm behaviour was not reported as an outcome |
| Sebastian, C., & Beer, M. D. (2005). Physical health of psychiatric patients admitted to a low secure challenging behaviour unit. *Journal of Psychiatric Intensive Care*, *1*(2), 77-83. | Dual-harm behaviour was not reported as an outcome |
| Selenius, H., Leppänen Östman, S., & Strand, S. (2016). Self-harm as a risk factor for inpatient aggression among women admitted to forensic psychiatric care. *Nordic journal of psychiatry*, *70*(7), 554-560. | No eligible psychological factors reported in relation to the dual-harm group |
| Szmukler, S. S. S. G. (1998). How predictable is violence and suicide in community psychiatric practice?. *Journal of Mental Health*, *7*(4), 393-401. | Dual-harm behaviour was not reported as an outcome |
| Sigafoos, J. (1995). Factors associated with aggression versus aggression and self-injury among persons with intellectual disabilities. *Developmental Disabilities Bulletin*. | Sample under study had a developmental condition |
| Sjgafoos, J., Elkins, J., Kerr, M., & Attwood, T. (1994). A survey of aggressive behaviour among a population of persons with intellectual disability in Queensland. *Journal of Intellectual Disability Research*, *38*(4), 369-381. | Sample under study had a developmental condition |
| Sillito, C. L., & Salari, S. (2011). Child outcomes and risk factors in US homicide-suicide cases 1999–2004. *Journal of Family Violence*, *26*(4), 285-297. | No eligible psychological factors reported in relation to the dual-harm group |
| Simó-Pinatella, D., Font-Roura, J., Alomar-Kurz, E., Giné, C., Matson, J. L., & Cifre, I. (2013). Antecedent events as predictive variables for behavioral function. *Research in Developmental Disabilities*, *34*(12), 4582-4590. | Dual-harm behaviour was not reported as an outcome |
| Sinclair, S. J., Bello, I., Nyer, M., Slavin-Mulford, J., Stein, M. B., Renna, M., ... & Blais, M. A. (2012). The Suicide (SPI) and Violence Potential Indices (VPI) from the Personality Assessment Inventory: A preliminary exploration of validity in an outpatient psychiatric sample. *Journal of Psychopathology and Behavioral Assessment*, *34*(3), 423-431. | Dual-harm behaviour was not reported as an outcome |
| Singh, N. N., Lancioni, G. E., Winton, A. S., Molina, E. J., Sage, M., Brown, S., & Groeneweg, J. (2004). Effects of Snoezelen room, Activities of Daily Living skills training, and Vocational skills training on aggression and self-injury by adults with mental retardation and mental illness. *Research in developmental disabilities*, *25*(3), 285-293. | Sample under study had a developmental condition |
| Singh, S. P., Santosh, P. J., Avasthi, A., & Kulhara, P. (1998). A psychosocial study of ‘self‐immolation’in India. *Acta Psychiatrica Scandinavica*, *97*(1), 71-75. | Dual-harm behaviour was not reported as an outcome |
| Slade, K. (2019). Dual harm: the importance of recognising the duality of self-harm and violence in forensic populations. | Not a quantitative study |
| Smucker, S., Kerber, R. E., & Cook, P. J. (2018). Suicide and additional homicides associated with intimate partner homicide: North Carolina 2004–2013. *Journal of urban health*, *95*(3), 337-343. | Dual-harm behaviour was not reported as an outcome |
| Stanley, B., Michel, C. A., Galfalvy, H. C., Keilp, J. G., Rizk, M. M., Richardson-Vejlgaard, R., ... & Mann, J. J. (2019). Suicidal subtypes, stress responsivity and impulsive aggression. *Psychiatry research*, *280*, 112486. | Dual-harm behaviour was not reported as an outcome |
| Swinton, M., & Hopkins, R. (1996). Violence and Self-injury. *Journal of Forensic Psychiatry*, *7*(3), 563-569. | Dual-harm behaviour was not reported as an outcome |
| Swogger, M. T., Walsh, Z., Maisto, S. A., & Conner, K. R. (2014). Reactive and proactive aggression and suicide attempts among criminal offenders. *Criminal justice and behavior*, *41*(3), 337-344. | Dual-harm behaviour was not reported as an outcome |
| Thiblin, I., Runeson, B., & Rajs, J. (1999). Anabolic androgenic steroids and suicide. *Annals of clinical Psychiatry*, *11*(4), 223-231. | Case study |
| Totsika, V., Toogood, S., Hastings, R. P., & Lewis, S. (2008). Persistence of challenging behaviours in adults with intellectual disability over a period of 11 years. *Journal of Intellectual Disability Research*, *52*(5), 446-457. | Dual-harm behaviour was not reported as an outcome |
| Vaughn, M. G., Salas-Wright, C. P., Underwood, S., & Gochez-Kerr, T. (2015). Subtypes of non-suicidal self-injury based on childhood adversity. *Psychiatric quarterly*, *86*(1), 137-151. | Dual-harm behaviour was not reported as an outcome |
| Velopulos, C. G., Carmichael, H., Zakrison, T. L., & Crandall, M. (2019). Comparison of male and female victims of intimate partner homicide and bidirectionality—an analysis of the national violent death reporting system. *Journal of trauma and acute care surgery*, *87*(2), 331-336. | Dual-harm behaviour was not reported as an outcome |
| Violanti, J. M. (2007). Homicide-suicide in police families: Aggression full circle. *International Journal of Emergency Mental Health*, *9*(2), 97. | No eligible psychological factors reported in relation to the dual-harm group |
| Virkkunen, M., De Jong, J., Bartko, J., & Linnoila, M. (1989). Psychobiological concomitants of history of suicide attempts among violent offenders and impulsive fire setters. *Archives of General Psychiatry*, *46*(7), 604-606. | No eligible psychological factors reported in relation to the dual-harm group |
| Weeke, A., & Oberwittler, D. (2018). A comparison of note writers and no note writers in homicide-suicide cases in Germany. *Archives of suicide research*, *22*(1), 11-19. | No eligible psychological factors reported in relation to the dual-harm group |
| Whitters, A. C., Cadoret, R. J., Troughton, E., & Widmer, R. B. (1987). Suicide attempts in antisocial alcoholics. *Journal of Nervous and Mental Disease*. | Dual-harm behaviour was not reported as an outcome |
| Yip, P. S., Wong, P. W., Cheung, Y. T., Chan, K. S., & Beh, S. L. (2009). An empirical study of characteristics and types of homicide–suicides in Hong Kong, 1989–2005. *Journal of Affective Disorders*, *112*(1-3), 184-192. | No eligible psychological factors reported in relation to the dual-harm group |

Appendix E. Risk of bias assessment

| Reference | Unbiased selection of cohort | Selection minimises baseline differences in demographic factors | Sample sizes calculated or minimum number > 1000 | Adequate description of sample | Validated methods for assessing predictor variables | Validated methods for ascertaining harmful behaviours | Blind to diagnostic, clinical, or participant status | Adequate follow up period | Missing data is minimal or adequately handled | Analysis controls for confounding | Analytical methods appropriate | Overall risk of bias |
| --- | --- | --- | --- | --- | --- | --- | --- | --- | --- | --- | --- | --- |
| **H-S studies** | | | | | | | | | | | | |
| **Benetiz-Borrego et al., 2013** | N | N | N | Partial | Partial | Y | N/A | N/A | Cannot tell | N | Y | High |
| **Flynn et al., 2009** | Y | N | Y^a^ | N | Partial | Y | N/A | N/A | Cannot tell | N | Y | Moderate |
| **Fridel, & Zimmerman, 2019a** | Y | N | Y^a^ | Y | Cannot tell | Y | N/A | N/A | Y | Y | Y | Low |
| **Fridel & Zimmerman, 2019b** | Y | N | Y^a^ | Y | Cannot tell | Y | N/A | N/A | Y | N | Y | Low |
| **Friedman et al., 2008** | N | N | N | Partial | Cannot tell | Y | N/A | N/A | Cannot tell | N | Y | High |
| **Haines et al., 2010** | Y | Y | N | Partial | Partial | Y | N/A | N/A | Cannot tell | N | Y | Moderate |
| **Heron, 2017** | N | N | N | Partial | Partial | Y | N/A | N/A | Cannot tell | Y | Y | Moderate |
| **Kalesan et al., 2018** | Y | N | Y^a^ | Y | Cannot tell | Y | N/A | N/A | Cannot tell | Y | Y | Low |
| **Leveillee et al., 2007** | Y | N | N | N | Cannot tell | Y | N/A | N/A | Cannot tell | N | Y | Moderate |
| **Liem et al., 2009** | N | Partial | N | Partial | Partial | Y | N | N/A | Cannot tell | N | Y | High |
| **Liem & Roberts, 2009** | N | N | N | Partial | Partial | Partial | N/A | N/A | Cannot tell | N | Y | High |
| **Logan et al., 2008** | Y | N | Y^a^ | Partial | Cannot tell | Y | N/A | N/A | Cannot tell | Y | Y | Moderate |
| **Logan et al., 2019** | Y | N | Y^a^ | Y | Cannot tell | Y | N/A | N/A | Cannot tell | Y | Y | Low |
| **Vatnar et al., 2019** | Y | N | N | Partial | Partial | Y | N/A | N/A | Cannot tell | Y | Y | Moderate |
| **Zimmerman & Fridel, 2020** | Y | N | Y^a^ | Y | Cannot tell | Y | N/A | N/A | Cannot tell | Y | Y | Low |
| **Total that met criteria** | 10/15 | 1/15 | 7/15 | 5/15 | 0 | 14/15 | 0/1 | N/A | 2/15 | 7/15 | 15 |  |
| **Non-H-S studies** | | | | | | | | | | | | |
| Ghossoub et al., 2018 | Y | N | Y^a^ | Y | N | N | N/A | N/A | Y | Y | Y | Low |
| Harford et al., 2014 | Y | N | Y^a^ | Y | Y | N | N/A | N/A | Cannot tell | Y | Y | Low |
| Harford et al., 2018a | Y | N | Y^a^ | Y | Y | N | N/A | N/A | Cannot tell | Y | Y | Low |
| Harford et al., 2018b | Y | N | Y^a^ | Y | Cannot tell | N | N/A | N/A | Cannot tell | Y | Y | Moderate |
| Hemming et al., 2021 | N | N | Y | Partial | Y | N | N | N/A | Y | Y | Y | Moderate |
| Hillbrand, 1992 | Cannot tell | N | N | Y | Partial | Y | N/A | N/A | Cannot tell | N | Y | Moderate |
| Huang et al., 2022 | Y | N | N | Partial | Partial | Y | N | N/A | Y | N | Y | Moderate |
| Laporte et al., 2017 | Y | N | N | Partial | Y | Cannot tell | N | N/A | Cannot tell | N | Y | Moderate |
| Lidberg et al., 2000 | Cannot tell | N | N | N | Y | Cannot tell | N | N/A | Cannot tell | N | Y | High |
| Richmond-Rakerd et al., 2019 | Y | N | Y^a^ | Partial | Partial | Partial | N/A | Y | Y | N | Y | Moderate |
| Stålenheim, 2001 | Cannot tell | N | N | Partial | Y | Y | N | N/A | Y | N | Y | Moderate |
| Steeg et al., 2019 | Y | N | Y^a^ | Partial | Partial | Y | N/A | N/A | Cannot tell | Y | Y | Low |
| Steinhoff et al., 2022 | Y | N | Y^a^ | Partial | Partial | N | N | Y | Y | Y | Y | Low |
| Swogger et al., 2011 | Cannot tell | N | N | Y | Partial | N | N/A | Y | Y | Y | Y | Moderate |
| Tardiff, 1981 | Y | N | N | Partial | Y | Y | N | N/A | Cannot tell | N | Cannot tell | Moderate |
| Watkins et al., 2017 | Cannot tell | N | Y^a^ | Partial | Y | Partial | N | N/A | Y | N | Y | Moderate |
| Hemming et al., 2021 | N | N | Y | Partial | Y | N | N | N/A | Y | Y | Y | Moderate |
| **Total that criteria met** | 9/16 | 0/16 | 7/16 | 6/16 | 8/16 | 5/16 | 0/8 | 3/3 | 8/16 | 8/16 | 15/16 |  |
